# Balanced informational feedback as part of an implementation/deimplementation bundle to support deimplementation efforts in antimicrobial stewardship: results from a mixed methods, hybrid type III implementation/effectiveness trial

**DOI:** 10.3389/fpubh.2026.1893058

**Published:** 2026-09-23

**Authors:** Westyn Branch-Elliman, Marlena H. Shin, Samuel Golenbock, Rebecca P. Lamkin, Dipandita Basnet, Judith M. Strymish, Dimitri Drekonja, Maria Rodriguez-Barradas, Teena Xu, Kathryn Colborn, Marin L. Schweizer, A. Rani Elwy, Hillary J. Mull

**Affiliations:** 1 Department of Medicine, Section of Infectious Diseases, VA Greater Los Angeles Healthcare System, Los Angeles, CA, United States; 2 Center for the Study of Healthcare Innovation, Implementation, and Policy (CSHIIP), Greater Los Angeles VA Medical Center, Los Angeles, CA, United States; 3 Department of Medicine, UCLA David Geffen School of Medicine, Los Angeles, CA, United States; 4 Center for Health Optimization and Implementation Research (CHOIR), VA Boston Healthcare System, Boston, MA, United States; 5 Section of Infectious Disease, VA Boston Healthcare System, Boston, MA, United States; 6 Department of Medicine, Harvard Medical School, Boston, MA, United States; 7 Department of Medicine, Section of Infectious Diseases, Minneapolis VA Healthcare System, Minnesota, MN, United States; 8 Department of Medicine, Section of Infectious Diseases, Michael E. DeBakey VA Medical Center, Houston, TX, United States; 9 Department of Medicine, Baylor College of Medicine, Houston, TX, United States; 10 University of Colorado School of Medicine, Aurora, CO, United States; 11 Department of Medicine, William S. Middleton VA Hospital, Madison, WI, United States; 12 Department of Medicine, University of Wisconsin-Madison, Madison, WI, United States; 13 Center for Health Optimization and Implementation Research (CHOIR), Bedford VA Medical Center, Bedford, MA, United States; 14 Department of Psychiatry, Brown University School of Medicine, Providence, RI, United States; 15 Department of Surgery, Boston University Chobanian & Avedisian School of Medicine, Boston, MA, United States

**Keywords:** implementation science, antimicrobial stewardship, hybrid trial, i-PARIHS, deimplementation, implementation/deimplementation bundle, mixed methods, cardiovascular implantable electronic device

## Abstract

**Background:**

Effective de-implementation approaches to reduce low-value or harmful care are limited. Prolonged antimicrobial use after skin closure is ineffective and increases harm but is nonetheless common following cardiovascular implantable electronic device (CIED) procedures. The aim of this quasi-experimental Implementation/Effectiveness study was to use an implementation/de-implementation (I/D) bundle of strategies to promote de-implementation of guideline-discordant prolonged antimicrobial use and improve outcomes.

**Methods:**

Hybrid III Implementation/Effectiveness study grounded in learning/unlearning theory and guided by the integrated-Promoting Action on Research Implementation in Health Services (i-PARIHS) framework with a non-randomized parallel group trial design (3 intervention and 8 control sites) over a 12-month period with an additional 12-months of follow up to assess sustainment. The I/D bundle included: blended facilitation, local champions, provider education, and access to locally-customized audit-feedback data about facility-level guideline compliance with benchmarking, CIED infections, and antimicrobial harms (acute kidney injury, allergic events, *Clostridiodes difficile* infections). Implementation outcomes were assessed quantitatively using a difference-in-differences model and qualitatively; clinical outcomes were assessed quantitatively.

**Results:**

2,293 CIED procedures were performed at the 3 intervention sites and 5,273 at the 8 matched control sites. Following I/D bundle intervention, rates of guideline non-compliance fell in the intervention sites but not the controls (OR, 0.16, 95% CI, 0.12–0.21). There was no change in CIED infections following the intervention (OR, 0.96, 95% CI, 0.48–1.93, *p* = 0.91) and a trend toward a reduction in composite antimicrobial harms (OR = 0.59; 95% CI: 0.34, 1.02, *p*-value = 0.059). Improvements were sustained during the 12-month period after active implementation and associated with a decrease in composite harm (aOR = 0.57; 95% CI: 0.34–0.91, *p* = 0.02). Strategies identified as effective for promoting de-implementation included education, locally-customized dashboard audit-feedback data with benchmarking to high-performing sites and feedback about both harm and benefit, blended facilitation, and provider’s readiness to change. Review and updating of hospital factors that promote sustainment of ineffective care, such as automated order sets, were also important. Practices learned and introduced from other practice settings were important barriers to change.

**Conclusion:**

The I/D bundle that included audit-feedback about patient-level harms of guideline discordant care was an effective strategy for reducing low-value care and improving quality.

**Trial registration:**

ClinicalTrials.Gov: NCT05020418.

## Highlights

**What Was Known Before this Study:**

- Low-value, ineffective, and harmful care is common in healthcare settings yet evidence-based interventions and implementation/de-implementation strategies to support improvement are limited.
- Barriers to de-implementation include liability concerns, risk-loss aversion and information silos and feedback loops that limit provider knowledge about harms.

**Added Value of this Study:**

- In this Hybrid III, mixed methods, multi-site trial, we demonstrate that an implementation/deimplementation bundle of strategies that included surveillance about patient-level harms (to address information loops and silos) with benchmarking (to address concerns about liability) reduced ineffective antimicrobial use and improved clinical outcomes.
- Reducing inappropriate antimicrobial use resulted in improved clinical outcomes by reducing antimicrobial-attributable adverse events. Cardiac device infections did not increase.
- The inclusion of balanced informational feedback about harms of continuing the low-value practice with reassurance that practice change did not lead to an increase in infections or liability was perceived as an important aspect of the successful deimplementation effort.

**Implications of All of the Available Evidence:**

- Information silos and feedback loops are important drivers of low-value care. Audit-feedback mechanisms to close these silos is an important strategy for supporting de-implementation efforts.
- Improving uptake of clinical guidelines supporting shorter durations of antimicrobial use following device implantations lead to improved clinical outcomes.

## Background

Implantation and utilization of cardiovascular implantable electronic devices (CIEDs) is increasing as the population ages (1). This uptick in usage is coupled with a simultaneous rise in the rate of CIED infections, which are highly morbid and often require complete device removal (2–5). The high morbidity and mortality associated with CIED infections place great importance on optimizing prevention at the time of the procedure, which may include methicillin-resistant *Staphylococcus aureus* decolonization efforts, skin cleaning, use of device pockets, and antimicrobial prophylaxis.

Antimicrobial prophylaxis given prior to skin closure is guideline-endorsed to prevent surgical and procedure associated infections and reduces post-intervention CIED infections (5–9). Antimicrobial prophylaxis continued after skin closure does not reduce post-procedural infections. Retrospective studies consistently demonstrate that prolonged prophylaxis does increase risk of antimicrobial-associated adverse events, including *Clostridiodes difficile* infections and acute kidney injuries (AKIs) (10, 11). Despite this harm-benefit profile strongly favoring immediate discontinuation of antimicrobials after skin closure, use of prolonged antimicrobial prophylaxis lasting for several days following CIED placements is common (12, 13). Provider-reported reasons for continuing the practice include legal and liability concerns about not providing accepted standard of care and, should the patient develop a subsequent infection, concerns about lack of systems for monitoring CIED infections and therefore limited ability to detect increases in infectious complications if practice changes are adopted, beliefs about the effectiveness of prolonged antibiotics, and factors that occur from other parts of the health system (e.g., order sets, other providers) that encourage them to continue, even if they recognize it is not evidence-based (14). Of note, surveillance requests from providers in the context of supporting de-implementation of ineffective antimicrobial use is not for the purposes of external reporting or evaluation; the infection surveillance information is requested as a mechanism for identifying an increase in infections following the policy change and for the purposes of *reassuring* the providers that harm did not arise as a result of reduced antimicrobial use. Factors associated with positive de-implementation practice change include support from local champions and thought leaders who are trusted and respected by the provider, and moving to a new practice setting with different standard processes (14).

De-implementation science focuses on reducing the use of interventions that are low-value, no longer effective, or ineffective (15). Although reduction in low-value care is recognized as critical, evidence-based strategies to support de-implementation efforts are limited. Due to the inherent human bias toward acts of commission rather than omission (e.g., a desire to do something) and a strong instinct opposing perceived losses, many evidence-informed models for de-implementation efforts focus on removal and replacement strategies (16, 17). Another theoretical approach to de-implementation is grounded in learning and unlearning theory (18, 19). Under this paradigm, de-implementation efforts should be viewed as a two-phased process, in which individuals and systems must learn to stop doing one thing (unlearning) and simultaneously learn to do another (learning).

In this paper, we report the main findings of our informatics-based, real-time audit and feedback intervention as part of a multi-faceted implementation/deimplementation (I/D) bundle of strategies to de-implement inappropriate antibiotic prescribing while promoting appropriate prescribing behavior.

## Methods

*Overview*: This hybrid type III implementation-effectiveness study was conducted at three large-volume Department of Veterans Affairs (VA) medical centers from March 2022 to November 2024. The study protocol was previously published (20–23). In brief, this study was initially designed as a prospective, stepped wedge non-randomized trial of the implementation/de-implementation (I/D) bundle intervention at three sites, but we were able to enhance the design to include eight contemporaneous control sites, making this a quasi-experimental prospective parallel group trial. De-implementation efforts and strategies were grounded in learning/unlearning theory and guided by the integrated-Promoting Action on Research Implementation in Health Services (i-PARIHS) framework (24). Barriers and facilitators to de-implementation were mapped to evidence-informed implementation strategies to create the I/D bundle intervention (25). The I/D bundle intervention was prospectively rolled out at each of the three intervention sites with approximately 3-month lags. De-implementation outcomes were assessed both qualitatively and quantitatively and clinical outcomes were assessed quantitatively. Sustainment of practice change and impact on clinical outcomes were also evaluated 12 months after the active facilitation period ended.

*Design*: Hybrid type III implementation/effectiveness quasi-experimental parallel group trial with pre-test/post-test parallel group stepped-intervention implementation and a qualitative summative evaluation with key stakeholders at the three intervention sites leading to an explanatory, sequential mixed methods analysis (Figure 1). Aligned with Hybrid III designs, the primary outcome was an implementation outcome (uptake of the evidence-based practice). Clinical outcomes were also assessed.

**Figure 1:**
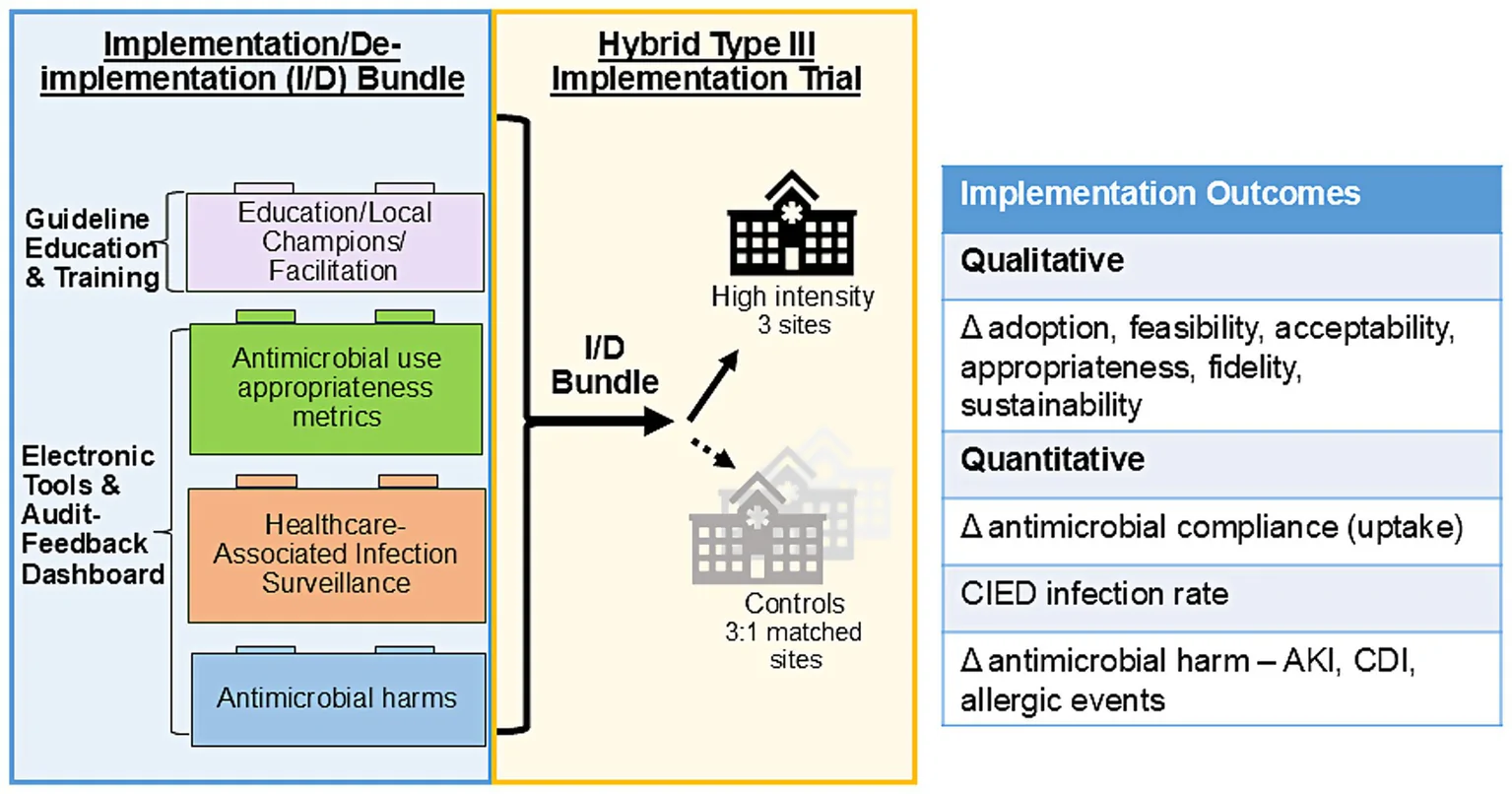
Overview of implementation/deimplementation bundle and hybrid III study design.

*Sites*: The three I/D bundle intervention sites were selected based on their high volume of procedures and high rates of guideline discordant practices at the time of study initiation. Control sites were selected using the greedy matching algorithm from the SAS macro %gmatch (26). A maximum of 3 control sites were matched to each study site based on total procedure volume during the study period and the facility rate of prolonged prophylaxis at baseline (e.g., 6 months prior to bundled implementation intervention at any of the participating intervention sites, quarters one and two of fiscal year 2022). Matching calipers (maximum absolute difference of 300 procedures for total volume and 25 percentage-points for baseline prophylaxis rate) were pre-specified prior to running the matching algorithm and the results were used as generated.

*I/D Bundle Intervention*: Intervention sites each received the I/D bundle, which included an educational intervention for providers (Supplementary materials), informational materials for patients (27), external facilitation provided by the study team in the form of implementation support, dashboard maintenance and adjustment and infection surveillance activities, internal facilitation provided by a local champion and trusted thought leader, and monthly audit-feedback reports provided via a secure dashboard about rates of guideline discordant (inappropriate) antimicrobial use, clinical outcomes (e.g., rates of CIED infection, rates of *C. difficile* infection, rates of AKI), and rates of allergic adverse events). Deidentified information about guideline concordance and outcomes at other, non-participating sites were also available for the purposes of benchmarking against high-performing facilities. Local adaptation for the educational intervention was permitted and the dashboard was developed and iteratively updated according to feedback from the intervention sites during the active facilitation period. Access to the dashboard was given to the local stewardship champion and to any other stakeholders at the site who requested access (e.g., cardiology chief, electrophysiologists, antimicrobial stewardship pharmacists). The audit-feedback dashboard was updated on a monthly basis and reminders were sent by the core study team to the local champions when dashboard updates were made. CIED infections flagged by the artificial-intelligence (AI)-facilitated algorithm underwent manual review by a member of the external facilitation team and findings from the manual review were posted to the dashboard in line-list format to assist with site-level infection prevention surveillance activities. The blended facilitation occurred for a minimum of 12 months at each of the intervention facilities and practice changes were assessed during and after the active blended facilitation process. A deidentified view of the final dashboard is available in Supplementary materials.

*Control and Follow Up Period:* The pre-intervention period was defined as the 1-year prior to the earliest intervention start date (March 2023) for all intervention sites and the follow-up period was 1-year after the latest intervention start (November 2023). Matched controls were included for the same time frame as their matched site. In an exploratory analysis, the follow up period was extended for an additional 1-year to evaluate sustainability of the practice change following the active implementation period.

*Outcomes*: All quantitative outcomes were assessed using the electronic health record (EHR) and methods of ascertainment were the same at intervention and control sites. The primary implementation outcome was facility-level rate of guideline-discordant antimicrobial use (e.g., uptake of evidence-based practice), defined as the number of cases with prolonged antimicrobial use/total number of procedures performed at the facility. Although more recent guidelines recommend immediate discontinuation following skin closure, earlier guidelines recommended discontinuation within 24 h following the end of the procedure. Given the study timeline, guideline discordance was defined as antimicrobials administered for >24 h following skin closure. Cases with infections at the time of the procedure or antimicrobials lasting >14 days following the procedure were presumed to be receiving treatment for active infections, and therefore not flagged for guideline-discordant use.

The main clinical outcome was the incidence of CIED infections within 90 days following the procedure, measured using previously published and validated AI-facilitated CIED infection flagging algorithm (28–30). Secondary clinical outcomes were also measured using variables extracted from the EHR and included a composite harms outcome that included: 7-day incidence of AKI as defined by the Kidney Injury Network, 60-day incidence of *C. difficile* infections, and 45-day incidence of antimicrobial-associated allergic events. Antimicrobial adverse events were defined as previously published (11, 31).

*Statistical Analysis*: Descriptive statistics are presented to assess the unadjusted rates and rate changes for each outcome between pre- and post-intervention. The primary statistical analysis used a difference-in-differences approach to assess the change in odds of each outcome measure for patients from facilities that received the intervention relative to those from matched control sites that did not receive the intervention during the one-year period before and after initiation of the intervention at each study site. Outcome measures were modeled using logistic regression with a random intercept to account for facility correlations. Unadjusted models included an indicator for “intervention site” (vs. control site), an indicator for “intervention initiation” time period (vs. pre-intervention period), and an interaction term. Covariate-adjusted models included variables for age, sex, race and ethnicity, and the Elixhauser comorbidity indices for 30-day hospital readmission and mortality (32). Because controls were temporally matched to intervention sites, we did not include time in the model. The post-intervention cutoff was defined as the date of the initial provider presentation for each facility. Control sites were assigned the post-intervention cutoff date of their respective matched study site. A secondary analysis assessed the sustainment of the intervention and impacts during the one-year period after study end. To assess the comparability of pre-intervention trends between intervention and control sites, we fit an exploratory logistic regression model restricted to the pre-intervention time period, modeling our primary outcome on “intervention site,” “study time” (months relative to each site’s respective intervention start date), and their interaction. All data extraction and analyses were conducted using SQL Server and SAS Enterprise Guide 8.3 (SAS Institute, Cary NC).

*Power Calculation (Implementation Uptake):* For the original stepped-wedge study design, we used the power estimation procedure of Hussey and Hughes (33), and determined that we would need 135 cases from each site to have 80% power to detect a 15% difference between intervention and control periods for our primary implementation outcome (e.g., uptake of guideline-concordant care). With our updated quasi-experimental design including additional control sites in a prospective, parallel group trial, we re-estimated power and determined we had >90% power to detect a 15% difference between the intervention and control sites over time (i.e., difference in slopes, or difference-in-differences), accounting for 300 procedures per period per site across 11 sites.

### Qualitative methods

#### Data collection

Between July 2024 and March 2025, semi-structured qualitative interviews were conducted via Microsoft Teams with key stakeholders at the three intervention sites as part of a summative evaluation. Our goal was to recruit and interview purposively selected key stakeholders who could provide us with salient information about experiences with and perceptions about the I/D Bundle Intervention as well as what helped lead to positive practice change and adoption of guideline concordant antimicrobial prescribing among the electrophysiology providers. Key stakeholders included electrophysiology team members, antimicrobial stewardship specialists/clinical pharmacists, and infectious diseases/infection control specialists (i.e., local champions and internal facilitators). The interviews focused on obtaining high “information power” from a smaller sample size of individuals most central to the intervention to ensure sufficient saturation across sites (34, 35).

An experienced qualitative researcher (MS) along with a research coordinator (DB) conducted the virtual interviews using a semi-structured interview guide, which consisted of questions that encompassed constructs of the i-PARIHS Framework: Innovation, Recipients, Context, and Facilitation and Proctor’s implementation outcomes (24, 36). The interview guide was designed to understand what components of the intervention were most effective, how the intervention impacted their clinical practice decisions, and potential future adaptations. Interview questions mapped to the i-PARIHS Framework are shown in Table 1. Interviews with electrophysiology team members lasted about 30 min while interviews with antimicrobial stewardship specialists/clinical pharmacists, and infectious diseases/infection control specialists lasted about 45 min.

**Table 1:** i-PARIHS constructs and interview questions.

i-PARIHS construct		Definitions (as adapted for this study)	Interview questions
Innovation (Deimplementation Bundle)	Evidence/underlying knowledge sources	Discussion about the evidence for the deimplementation bundle being implemented: Research/published guidelines; local practice information	What new information did you learn from the educational session/dashboard report? How did the educational session/dashboard report change your understanding about antibiotic risks?
	Degree of fit	Discussion about the extent to which the bundle is compatible with (1) the values and norms of individuals implementing the innovation and/or (2) the existing practices and operations of the setting (workflows, processes, etc.)	How did the educational session/dashboard report fit with/impact work flow?
	Usability	Discussion about the degree of ease/difficulty with which the bundle was adopted/used	What did you find useful/not useful about the educational session/dashboard report? How can the educational session/dashboard report be improved?
	Observable results	Discussion about the degree to which positive results of the bundle and practice change are directly observable	Did outcomes for your patients change? What drives your prescribing now?
Recipients	Personal attributes	Discussion of personal traits/characteristics of the electrophysiologists and their motivation for changing practice	How did the information in the educational session/dashboard report help to change your own prescribing practice? Were you able to use the information in the educational session/dashboard report to change the practice of others? What worked/did not work?
	Time, resources, support	Discussion about how sufficient time, resources and support affect the electrophysiologists to receive the deimplementation bundle	Were there any barriers experienced in receiving the deimplementation bundle?
Context	Leadership support	Discussion about characteristics or behaviors of local champions, section chiefs that either support or interfere with the implementation of the bundle	How did leadership support the implementation of the bundle?
	Culture	Discussion about the culture among electrophysiologists, including prevailing norms, values, beliefs	How did benchmarking change concerns about being “an outlier”? How did information about external practices change internal practices?
Facilitation	Internal/External	Discussion about the activities that local champions (internal facilitators) and the study team (external facilitators) performed to facilitate implementation of the bundle	How did the local champion impact prescribing practice decisions? Were the study team activities, as external facilitators, effective? Why/why not?

### Qualitative data analysis

A rapid qualitative analysis approach was used to summarize key transcript information (37). To conduct a directed content analysis (38), first, a codebook that contained definitions and examples of constructs from the i-PARIHS Framework (39) and implementation outcomes (36) was developed. Next, two qualitative analysts (MS, DB) reviewed one of the transcripts and summarized this information into a summary template; this process helped to establish agreement on the codebook definitions and examples as well as ensure that the summary template appropriately captured key information from the transcripts. Once the two analysts discussed this summarized transcript and consistency of definitions and examples was established, one of the analysts (DB) proceeded to summarize the interview transcripts using the summary template. Upon completion of all summary templates, that information was transferred into a matrix display to draw out key findings in an organized and efficient manner (40). The other qualitative analyst (MS) conducted a secondary review to ensure accuracy of the information in the summary templates and matrix display. Thereafter, the team conducted an analysis across participants to determine the key factors that affected the implementation of the intervention and practice change. Findings were discussed with the larger team regularly throughout the data analysis process to allow for broader input.

### Ethical considerations

This study was approved as exempt research by the VA Boston Research and Development committee with a waiver of informed consent and is registered in clinicaltrials.gov (NCT05020418; https://clinicaltrials.gov/study/NCT05020418?term=NCT05020418&viewType=Card&rank=1).

## Results

### Quantitative analysis

Three I/D bundle intervention facilities were matched to 8 temporally-matched control facilities with similar procedure volume and baseline rates of prolonged antimicrobial prophylaxis (See Table 2 for baseline descriptive details of sites). During the study period, 2,293 CIED procedures were performed at the 3 I/D bundle intervention sites vs. 5,273 at the 8 matched control sites (Figure 2, CONSORT). The median age of patients was 76 and 75 at the intervention and control sites, respectively. Patients from the intervention sites were more likely to have a military service-connected disability compared with those from control sites (72.2% vs. 67.4%). Patients were otherwise similar on sex, race and ethnicity, marital status and comorbidity scores across intervention and control groups.

**Table 2:** Patient characteristics of VHA cardiac device patients overall and by intervention vs. control site group March 2022 to November 2025.

	Intervention vs. Control site patients		
	Total (*n* = 5,469)	Study site patients (*n* = 1,694)	Control site patients (*n* = 3,775)
Post-intervention volume, n(%)	2,793 (51.1)	837 (49.4)	1956 (51.8)
Age, median (IQR)	75.0 (70.0,80.0)	76.0 (70.0,81.0)	75.0 (69.0,79.0)
Age groups, n(%)			
<60	393 (7.2)	108 (6.4)	285 (7.5)
60–69	921 (16.8)	259 (15.3)	662 (17.5)
70–79	2,755 (50.4)	846 (49.9)	1909 (50.6)
80–89	1,114 (20.4)	370 (21.8)	744 (19.7)
> = 90	286 (5.2)	111 (6.6)	175 (4.6)
Sex female, n(%)	153 (2.8)	39 (2.3)	114 (3.0)
Race/ethnicity, n(%)			
White	4,065 (74.3)	1,237 (73.0)	2,828 (74.9)
Black	796 (14.6)	299 (17.7)	497 (13.2)
Other (Hispanic)	166 (3.0)	51 (3.0)	115 (3.0)
Other (non-Hispanic)	90 (1.6)	25 (1.5)	65 (1.7)
Other (unknown)	352 (6.4)	82 (4.8)	270 (7.2)
Marital status, n(%)			
Married	3,025 (55.3)	895 (52.8)	2,130 (56.4)
Not married	431 (7.9)	163 (9.6)	268 (7.1)
Divorced/separated	1,499 (27.4)	489 (28.9)	1,010 (26.8)
Widowed	492 (9.0)	141 (8.3)	351 (9.3)
Service-connected disability, n(%)	3,624 (66.3)	1,178 (69.5)	2,446 (64.8)
Days on postoperative antibiotics, median (IQR)	4.0 (1.0,6.0)	3.0 (0.0,6.0)	4.0 (1.0,6.0)
Days on postoperative prophylaxis, median (IQR)	4.0 (1.0,6.0)	3.0 (0.0,6.0)	4.0 (1.0,6.0)
Hemodialysis for ESRD, n(%)	92 (1.7)	37 (2.2)	55 (1.5)
Elixhauser comorbidity index: 30-day readmission, median (IQR)^a^	10.0 (5.0,17.0)	10.0 (4.0,16.0)	10.0 (5.0,17.0)
Elixhauser comorbidity index: mortality, median (IQR)^b^	8.0 (0.0,16.0)	8.0 (0.0,16.0)	8.0 (0.0,16.0)
Comorbidity diagnoses, n(%)			
Cancer	831 (15.2)	262 (15.5)	569 (15.1)
Diabetes (with and without complications)	2,573 (47.0)	774 (45.7)	1799 (47.7)
Hypertension (complicated or uncomplicated)	4,378 (80.1)	1,291 (76.2)	3,087 (81.8)
Obesity	1,066 (19.5)	290 (17.1)	776 (20.6)
Heart failure	2,991 (54.7)	890 (52.5)	2,101 (55.7)
Renal failure	1,385 (25.3)	429 (25.3)	956 (25.3)
Valvular disease	969 (17.7)	336 (19.8)	633 (16.8)
Liver disease	384 (7.0)	120 (7.1)	264 (7.0)
Peripheral vascular disease	983 (18.0)	315 (18.6)	668 (17.7)
Chronic pulmonary disease	1,272 (23.3)	297 (17.5)	975 (25.8)

a Elixhauser comorbidity index for risk of 30-day all-cause hospital readmission.

b Elixhauser comorbidity index for risk of in-hospital mortality.

**Figure 2:**
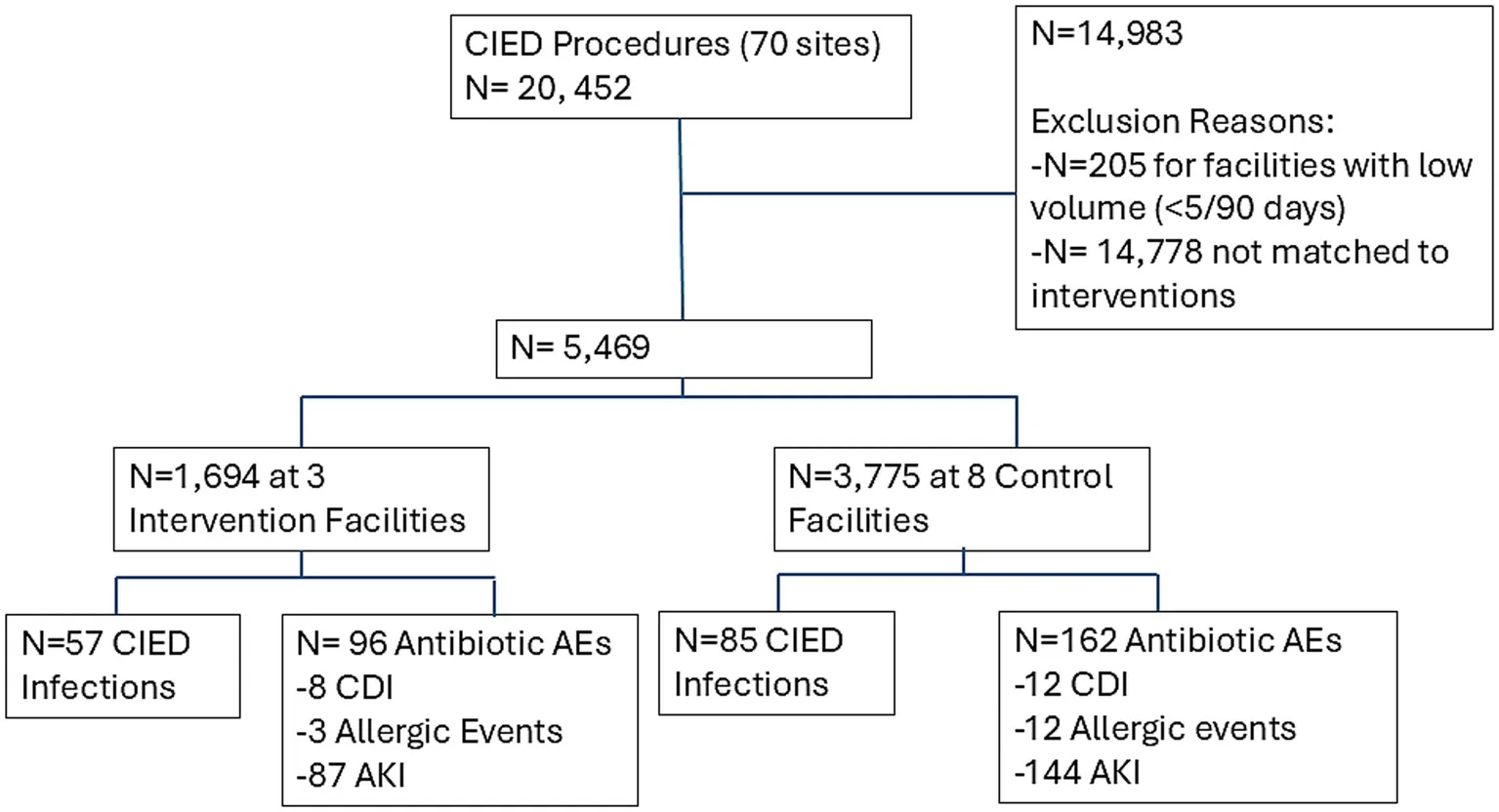
CONSORT flow of cardiovascular implantable electronic device (CIED procedures) and clinical outcomes among the three high-intensity implementation/deimplementation bundle sites and the eight matched control sites. CIED, cardiovascular implantable electronic device. AKI, acute kidney injury. All outcomes were assessed electronically. Sites were not randomized and control sites were selected using the greedy match procedure.

### Adaptations

Throughout the facilitated implementation, I/D bundle intervention sites were able to review data and provide feedback to the study team. Based on this feedback, several additions to the dashboard were made. Specifically, one site asked that duration of antibiotics following the procedure be included such that partial change (e.g., a reduction) could be identified and provided to the electrophysiologists. Another site asked for more frequent (monthly) updates of antimicrobial use during the active implementation phase to support more active audit-and-feedback during the early phase of the rollout. Additional adaptations included provider-level rates of guideline-concordant practice to direct individual feedback and updates to the definitions of guideline concordance based on local practices (specifically, to include longer durations of prophylaxis given a standard practice of >14 days of post-procedural use at one facility). Site engagement and fidelity to de-implementation strategies are presented in Table 3.

**Table 3:** Implementation/deimplementation strategy (I/D) bundle fidelity at the three intervention sites.

Deimplementation bundle element	Site 1	Site 2	Site 3
Educational sessions	1 (Delivered by local champion and study team collaboratively)	1 (Delivered by the local champion only)	2 (Two educational sessions conducted, first delivered by the local champion only; second session, 4 months later, delivered by the study team)
Internal champion support	One physician champion	One physician champion	One physician champion
Additional local stakeholder support	Antimicrobial stewardship pharmacist, cardiology chief, facility leadership	Antimicrobial stewardship pharmacist, infectious disease pharmacist, EP physician	Infection disease chief, antimicrobial stewardship pharmacist
Facilitation activities and frequency	1 collaborative educational session Local champion reviewed and monitored dashboard data trends monthly and updated EP physicians 3 bi-monthly side wide meeting	Initial discussion meeting: Local champion had a meeting with EP staff (MDs, APPs, RNs and schedulers) two months before the educational session Tailored educational session: Local champion customized to fit the specific needs of the site and secure providers buy-in Order set modification: Electrophysiology order set changed with discussion and support from EP Physicians and ID pharmacist champion 3 bi-monthly site-wide meeting	2 educational sessions conducted Local champion had discussion meetings with two EP physicians and email follow-ups with EP attendings. Monthly site-specific dashboard reports on post-procedure prophylaxis duration were shared by the study team and reviewed by the local champion, with updates provided to EP physicians every 2 months over a 12-month period. 3 bi-monthly site wide meeting
Dashboard access	Local physician champion	Local physician champion	Local physician champion and Infectious Disease Chief
Dashboard adaptation requests	Monthly reports, access for additional providers, provider-level reports	Access for pharmacist champion	Adaptation to local antimicrobial stewardship use, addition of duration metrics to reports
Patient education materials	Provided, printed but not used	Provided but not used	Provided but not used
Additional factors	Electrophysiologists full-time at facility with rotating trainees	Electronic order set present at study initiation Electrophysiologists full-time at facility with rotating trainees	Substantial proportion of electrophysiologists provided care at VA and other sites, including private facilities; rotating trainees

### Primary implementation outcome (uptake)

Prior to the bundled intervention, rates of guideline non-compliance at each of the three intervention sites ranged from 66.7 to 77.9%; baseline rates were similar at the control sites (range, 42.6 to 96.1%). Results from a pre-intervention sensitivity analysis showed a non-statistically significant interaction between site type and time (estimate = −0.0008; 95% CI:-0.037, 0.036; *p* = 0.966), supporting the comparability of pre-intervention trends between intervention and control groups. Results were similar for the clinical outcomes.

Following the bundled de-implementation intervention, rates of guideline non-compliance improved at two of the study sites but were unchanged at the third, resulting in an overall decrease of non-compliance in the three intervention sites of 44.8% between the pre- and post-intervention time periods (Figure 3). In contrast, all eight matched control sites demonstrated no observable post-intervention rate change. Adjusted logistic regression results showed a statistically significant marked reduction in the odds of receiving guideline discordant care following the intervention for procedures performed at facilities that received the intervention relative to those from matched control sites (adjusted odds ratio [aOR] = 0.16; 95% confidence interval [CI]: 0.12, 0.21); (Table 4).

**Figure 3:**
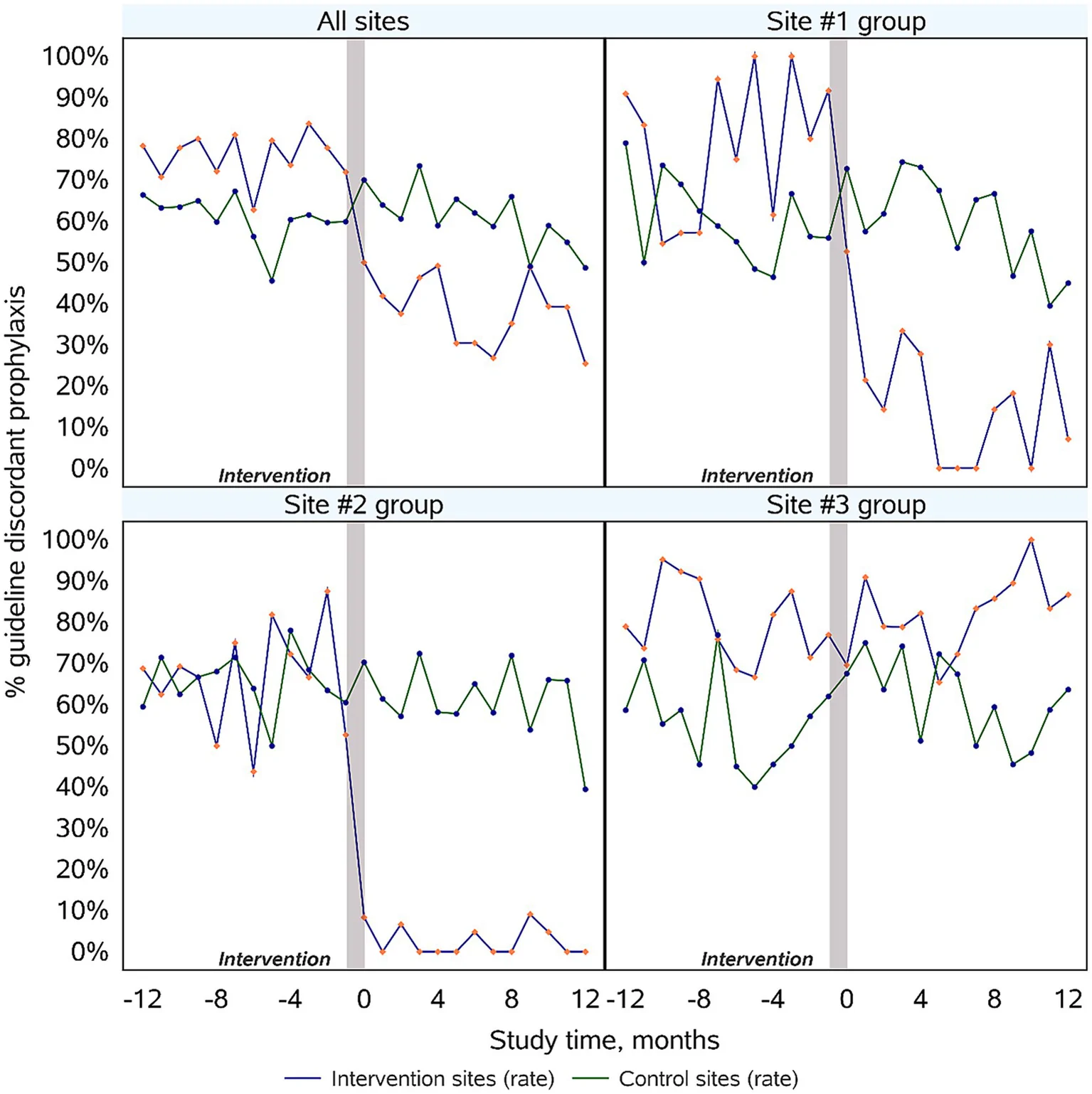
Rates of guideline-discordant antimicrobial prophylaxis at three study sites vs. controls. Primary implementation outcome (uptake) results. The three intervention sites are depicted in green and the control sites in blue. The gray line depicts the start of the active implementation at the control sites (e.g., intervention start). The first panel depicts summary results from all sites combined and subsequent panels present site-level outcomes with their associated matched controls.

**Table 4:** Unadjusted and covariate-adjusted model results assessing change in odds of primary and secondary outcomes for procedures from intervention sites relative to control sites (March 2022–November 2024).

Outcomes	Unadjusted odds ratio (OR)	Adjusted OR (95% CI)	*p*-value
Guideline-discordant prophylaxis^a^			
Time (Post-intervention)	0.89	0.89 (0.77–1.03)	0.110
Study site (vs. Control)	1.73	1.71 (0.41–7.16)	0.461
**Intervention Site (Post I/D Bundle)**	**0.16**	**0.16 (0.12–0.21)**	**<0.0001**
CIED predicted infection^b^			
Time (Post-intervention)	0.95	0.93 (0.60–1.44)	0.755
Study site (vs. Control)	1.47	1.54 (0.85–2.76)	0.153
**Intervention Site (Post I/D Bundle)**	**0.97**	**0.96 (0.48–1.93)**	**0.916**
Antimicrobial harms^a^^,^^c^			
Time (Post-intervention)	1.10	1.06 (0.76–1.47)	0.739
Study site (vs. Control)	1.68	1.74 (0.96–3.13)	0.066
**Intervention Site (Post I/D Bundle)**	**0.61**	**0.59 (0.34–1.02)**	**0.059**

Implementation and clinical outcomes at intervention sites are indicated in “Intervention Site Post I/D Bundle” and highlighted in bold.

a Adjusted models are adjusted for facility, age, sex, race/ethnicity and Elixhauser comorbidity indices.

b Adjusted models are adjusted for facility, age, sex and Elixhauser comorbidity indices.

c Antimicrobial harms composite outcome includes post-procedure acute kidney injury (AKI), C. difficile infection, and antimicrobial-related allergic adverse events.

### Clinical outcomes

Across the study period, facility CIED infection rates ranged from 0 to 4.7%. Prior to the I/D bundle intervention, 31 CIED infections occurred following 833 procedures (3.7%) at the intervention sites vs. 42 infections among 1758 procedures (2.4%) at the control sites (Table 5). Following the intervention, the rate of CIED infections remained similar at intervention and control sites (3.2 and 2.3% respectively). Results from the adjusted regression model showed no difference in the post-intervention odds of CIED infection between intervention and matched control sites (adjusted odds ratio [aOR] = 0.96; 95% CI: 0.48, 1.93, *p* = 0.91).

**Table 5:** Procedural volume, cardiovascular implantable electronic device infections, acute kidney injuries, *C. difficile* infections, and allergic events at the control and intervention sites during the pre and post-intervention periods.

	Total volume	CIED infections^a^	Acute kidney injuries^b^	*C. difficile* infections^c^	Allergic events^d^
Intervention sites (*N* = 3)	1,694	57	87	8	3
Pre	857	31	54	3	3
Post	837	26	33	5	0
Control sites (*N* = 8)	3,775	85	144	12	12
Pre	1,819	42	65	7	3
Post	1,956	43	79	5	9

a CIED, Cardiovascular Implantable Electronic Device Infection, defined by electronic flagging algorithm with a 90-day post-procedure surveillance period.

b Acute Kidney Injury defined based on Acute Kidney Injury Network definitions and laboratory-defined. Patients flagged as currently receiving renal replacement therapy were not counted in the ascertainment of this outcome.

c C. difficile infections within 60 days of the index procedure.

d Allergic events, defined according to the electronic flagging tool, within 45 days of the index procedure. Surveillance period to account for oral antibiotics and post-discharge use and event risk.

We identified 231 cases of AKI, 20 cases of *C. difficile*, and 15 cases of antimicrobial allergic events across the 11 sites. Prior to the I/D bundle intervention, the incidence of antimicrobial-associated harms was slightly higher at the intervention sites (6.8%) compared to those at control sites (4.0%). Following the I/D bundle intervention, the incidence of antimicrobial-associated harms decreased by 2.2% at intervention sites compared to an increase of 0.6% at control sites (2.9% difference in absolute risk reduction). The adjusted odds ratio showed a trend toward lower odds of experiencing an antimicrobial-associated harm at facilities following the intervention compared to facilities that did not receive the intervention (OR = 0.59; 95% CI: 0.34, 1.02, *p*-value = 0.059). In the exploratory analysis extending the study period for an additional 12 months, improvements in uptake of guideline concordant care persisted (Table 6). These sustained improvements were not associated with any increase in CIED infection (*p* = 0.92) but were associated with a decrease in antimicrobial-associated harms (aOR = 0.57; 95% CI: 0.34–0.91, *p* = 0.02).

**Table 6:** Unadjusted and covariate-adjusted model results assessing change in odds of primary and secondary outcomes for procedures from intervention sites relative to control sites, with extended follow-up period (March 2022–November 2025).

Outcomes	Unadjusted odds ratio (OR)	Adjusted OR (95% CI)	*p*-value
Guideline-discordant prophylaxis^a^			
Time (Post-intervention)	0.63	0.63 (0.56–0.72)	<0.0001
Study site (vs. Control)	1.87	1.80 (0.36–8.92)	0.471
*Intervention Site (Post I/D Bundle)*	0.21	0.21 (0.16–0.27)	<0.0001
CIED predicted infection^b^			
Time (Post-intervention)	0.87	0.86 (0.60–1.25)	0.433
Study site (vs. Control)	1.25	1.31 (0.74–2.30)	0.352
*Intervention Site (Post I/D Bundle)*	1.01	0.97 (0.53–1.77)	0.925
Antimicrobial harms^a^^,^^c^			
Time (Post-intervention)	1.09	1.04 (0.76–1.44)	0.789
Study site (vs. Control)	1.98	2.11 (1.11–3.98)	0.022
*Intervention Site (Post I/D Bundle)*	0.57	0.56 (0.34–0.91)	0.020

Implementation and clinical outcomes at intervention sites are indicated in “Intervention Site Post I/D Bundle”.

a Adjusted models include effects for facility, age, sex, race/ethnicity and Elixhauser comorbidity indices.

b Adjusted models include effects for facility, age, sex and Elixhauser comorbidity indices.

c Antimicrobial harms composite outcome includes post-procedure acute kidney injury (AKI), c. difficile infection, and antimicrobial-related allergic adverse events.

### Qualitative results

Fourteen interviews were conducted across the three intervention sites. Six participants were electrophysiology team members (i.e., physician, nurse practitioner, section chief); four were antimicrobial stewardship specialists/clinical pharmacists; and four were infectious diseases/infection control specialists.

Five key factors were identified across the three I/D bundle intervention sites that helped lead to positive practice change and adoption of guideline concordant antimicrobial prescribing among the electrophysiology providers:

(1) Educational session presentation;
(2) Dashboard audit-feedback data;
(3) Internal facilitation through strong infectious disease champion support;
(4) External facilitation;
(5) Providers’ readiness to collectively make the practice change together.

The only component of the intervention that was not used by sites, and thus, was not a factor that helped lead to practice change, was the patient education handout (27). Table 7 presents these factors along with the relevant i-PARIHS constructs and representative quotes. While our primary implementation outcome is adoption, Table 8 presents the key findings for the other implementation outcomes of feasibility, acceptability, appropriateness, fidelity, and sustainability that were qualitatively-assessed for this study.

**Table 7:** Key i-PARIHS constructs, factors for adoption, and representative quotes.

Key i-PARIHS construct	Key factors for adoption of guideline concordant antimicrobial prescribing	Representative quote
Innovation-evidence/underlying knowledge sources	Educational session Dashboard data	“Antibiotic use is not risk-free. I feel like that was something [from the educational session] that stuck out in my mind.” *Site A, Participant 2* “People are willing to change, you just got to show them the studies. It’s really our whole system is evidence-based.” *Site B, Participant 1*
Innovation-usability	Educational session Dashboard data	“Data is compelling, that’s what shows where there’s a problem, how we compare, and then if things are improving or not… You can only argue so much against data.” *Site C, Participant 4*
Innovation-observable results	Dashboard data	“Having the [dashboard] information that what we have from our facility that [infection rate] has not increased was the important data for us… knowing that it wasn’t going to change the infection rate and so it made sense to change our practice.” *Site A, Participant 3*
Recipients-personal attributes	Dashboard data Providers’ collective readiness	“You want to be somewhere, look at your data and feel that you are happy where you are so, [the dashboard] is useful.” *Site A, Participant 4* “[D]ata in particular, I think, were the thing that were most convincing and appreciated…I could use the risk–benefit of having a long course [of antibiotics]…the benefit has to match up to the risk and I think that calculation of that awareness really is driven by data. I would say that was probably what motivated [practice change the] most.” *Site C, Participant 1*
Content-culture	Dashboard data Providers’ collective readiness	“Probably seeing what the other sites are doing. So, for the facility, like you mentioned, does seeing what other sites are doing, will that affect your clinical practice? I think that makes a huge difference.” *Site A, Participant 5* “[W]e have been trying to change the duration for the longest time, and for the first time, [electrophysiology providers] agreed as a group that they were going to decrease the number of days.” *Site C, Participant 4*
Facilitation	Internal External	“[The ID champion/Internal facilitator’s] definitely been a strong advocate for decreasing antibiotic use and [a] prominent clinician on the service and deals with the consequences of patients with infections and I think that’s important to have someone who has that perspective…it’s much easier working with someone who has to deal with the problems that arise with us rather than someone who is a bit disconnected or not necessarily part of the clinical team.” *Site A, Participant 2* “I think it was great getting the reports sent on a regular basis and having that support in that second session. Just hearing it from a different facility and different experience, I think, was very helpful.” *Site C, Participant 3*

**Table 8:** Other implementation outcomes, definitions, and key themes.

Implementation outcome	Definition (as adapted for this study)	Key themes
Feasibility	Extent to which the de-implementation bundle can be used	De-implementation bundle is mostly feasible to implement, although some logistical challenges occurred: ◦ Changes in providers’ schedules led to some variation in the educational session attendance than initially expected ◦ Constraints in time hindered providers’ ability to review the dashboard reports on a regular basis
Acceptability	Perception that the de-implementation bundle is agreeable	Quality and strength of the evidence presented during the educational session and the data in the dashboard reports led to the acceptability of practice change Local ID champions are trusted site experts whose facilitation activities were instrumental to providers’ receptivity and motivation in making the practice change
Appropriateness	Perceived fit of the de-implementation bundle	Patient materials did not fit within workflow given already existing available materials for distribution to patients for pre- and post-procedure care Informational materials for patients were the least relevant in motivating or helping lead to practice change
Fidelity	Degree to which the de-implementation bundle is implemented as planned	Educational session, dashboard reports, and facilitation were delivered as planned but informational materials for patients were not distributed Providers adhering to guideline-concordant antimicrobial prescribing for the most part once decision to make practice change occurred Ongoing reminders about the guidelines are needed, particularly when providers rotate through the VA and have affiliations external to the VA as practices may not be consistent across settings
Sustainability	Extent to which guideline concordant antimicrobial prescribing becomes part of normal practice	Guideline concordant prescribing practices are likely sustainable given that providers now understand and are more aware of: ◦ The evidence and harms associated with antimicrobial use ◦ The data that support decrease in post-procedure antimicrobial use

### Site-level impacts

Drivers of prolonged prophylaxis were different at the three I/D bundle intervention sites, resulting in varied actions and mechanisms of practice change. At the first intervention site, change was mediated through building trust with the electrophysiologists during an early shared educational session with local infectious diseases leadership, cardiology leadership and the facility electrophysiologists coupled with dashboard access. An important driver of change was cardiology chief support for the intervention. Following the session, electrophysiologists reviewed evidence and requested local data and practice change occurred quickly, with one electrophysiologist’s practice change lagging the others but ultimately demonstrating improvement. Communications about support and plans for the change were made to the electrophysiologist nurse practitioners at the facility who also supported and facilitated the change. No specific orders sets had been created at this facility and thus there was not a need to make changes in the electronic health record.

At the second site, much prolonged prophylaxis was driven by long-standing discharge order sets embedded in the electronic health record that included a standard order for post-procedural antimicrobials. Following educational sessions, introduction of the dashboard, and leadership support and interest in de-implementing the practice, the order set was updated in the electronic health record to no longer recommend post-procedural doses. Elimination of the order set had an immediate and lasting impact on practices. Additionally, the site champion at the second site provided ongoing encouragement to cardiology stakeholders, including provision of feedback about low rates of CIED infections and a facility-level reduction in antimicrobial-associated harms.

Uptake of clinical guideline-recommended practice at the third site was limited, attributed to several factors. First, electrophysiologists at this site demonstrated limited engagement and support for the project, as evidenced by difficulty scheduling educational sessions with the entire group. Second, electrophysiologists at this site voiced skepticism about the planned practice change, citing protocols at other facilities where they also provided care. Third, electrophysiologists at this site indicated during educational sessions that their patients were more complex than at other sites, warranting more aggressive antimicrobial use strategies. There was also a perception that the antibiotics selected for prolonged prophylaxis were not harmful and therefore there was not an important reason to change them. However, despite these barriers, the electrophysiologists agreed to shorten the total duration of antimicrobials following the intervention, albeit not to the guideline-recommended level.

#### Impact of de-implementation bundle strategy elements: Educational session presentation

Across all sites, key stakeholders discussed how the educational session provided critical information, that electrophysiology providers were not aware of, about the potential consequences of prolonged antimicrobial prophylaxis through scientific evidence from research studies and clinical guidelines (i-PARIHS constructs *Innovation-Evidence/Underlying Knowledge Sources, Usability*). The rigor and quality of the scientific evidence from research studies and clinical guidelines were viewed as the main content areas of the presentation that helped to change electrophysiology providers’ perspectives. Key stakeholders discussed how they gained knowledge due to the research studies and clinical guidelines presented during the educational sessions, which raised their awareness about the lack of benefits and how data did not support extended antibiotics use post-procedure. In addition, key stakeholders highlighted how the dashboard demonstration presented during the educational session helped to emphasize the evidence from research studies and clinical guidelines, which provided them with a better understanding of how data did not support extended use of antibiotics post-procedure. Electrophysiology providers felt that the concrete data and visual graphs demonstrated that prolonged antibiotics use increases risks. The combination of scientific evidence and dashboard audit-feedback data presented during the educational session were highlighted as important learning components that helped to increase knowledge and awareness and motivate changes in electrophysiology providers’ personal practice.

One barrier to application of this de-implementation strategy noted by key stakeholders was the challenges in convening the electrophysiology providers together to attend the educational session due to conflicts in their schedules (i-PARIHS constructs *Innovation-Degree of fit, Recipients-Time*). In addition, trainees, who frequently place post-procedure orders and rotate in and out of the department, part-time providers, and other electrophysiology team members (e.g., nurses) were not consistently present during the educational sessions and thus reaching these end-users was inconsistent with an educational session scheduled once (or twice, as was done at one site). Stakeholders suggested that the educational session should be scheduled on a regular and recurring basis to ensure that information is communicated and shared more broadly and providers are aware of the most up-to-date guidelines and prescribing practices.

#### Audit-feedback dashboard with information about processes, outcomes, and healthcare-associated infection surveillance

Key stakeholders noted the dashboard is a user-friendly display that offered compelling data of several metrics, which had not been previously quantified or available at the intervention sites, for identifying areas of concern, benchmarking performance, and tracking improvement opportunities (i-PARIHS constructs *Innovation-Evidence/Underlying knowledge sources, Usability, Degree of fit*). The dashboard was viewed as an important tool that allowed for the continuous validation and reassurance of electrophysiology providers’ practices and offered a streamlined and comprehensive way of conducting surveillance activities given limited local resources. The ability to interact with the data in near real-time and access different layers of information were highlighted as advantages of the dashboard. In addition, stakeholders discussed how the dashboard was an important driver of practice change that encouraged providers to make informed decisions. The concrete audit-feedback and benchmarking data summarized and presented in the dashboard helped electrophysiology providers to see that clinical outcomes (e.g., infection rates) did not increase or change despite decreasing the antimicrobial prophylaxis duration. Furthermore, the comparative data of clinical outcomes across VA facilities helped to reassure electrophysiology providers of the practices that their colleagues used at other sites and alleviate their concerns of being “an outlier” (i-PARIHS constructs *Recipients-Personal attributes, Context-Culture)*.

Although electrophysiology providers offered positive feedback about the dashboard data and how it contributed to their practice change, they were unable to review this information on a regular basis due to time constraints. Thus, they were not as up to date on how their practice change had affected patient outcomes, although they understood that their practice change had led to positive outcomes (i-PARIHS constructs *Innovation-Observable results, Recipients-Time*). Stakeholders suggested that including a brief summary of the trends with the reports would allow the electrophysiology providers to review this information more efficiently, which also would help them to become better aware of how this practice change affects patient outcomes.

#### Facilitation (i-PARIHS constructs facilitation—internal, external)

There were five main strategies used by both local site Infectious Diseases (ID) champions and the study team to perform internal and external facilitation:

(1) Providing education/information;
(2) Collecting data/providing feedback;
(3) Building relationships;
(4) Problem identification and resolution;
(5) Planning/preparing for implementation;
(6) Providing administrative/technical support.

While overall the strategies were similar, specific internal and external facilitation activities varied.

#### Internal facilitation (strong ID champion and trusted expert support)

Overall, the ID champions, as internal facilitators, were a critical component in the implementation of the intervention. As trusted experts, they planned and prepared for the implementation of the intervention by tailoring the intervention to the local context and to fit the needs of the electrophysiology providers. In addition, the ID champions worked to build relationships, which increased engagement, buy-in, and support across multiple stakeholders at the sites. For the educational session, the ID champions helped to tailor the presentation by considering current local practices and determining what information would be needed to help electrophysiology providers consider moving forward to make practice changes. Their presence at this meeting also emphasized the importance of the intervention, which led to stronger buy-in among electrophysiology team members. ID champions regularly reviewed the dashboard data and provided feedback to and met with electrophysiology providers, as needed, to discuss trends in the data. ID champions also provided administrative support, such as organizing and scheduling meetings. Technical support included working directly with an expert who could update order sets and developing visuals to help interpret the dashboard data so that electrophysiology providers could better understand the trends, thereby increasing awareness of the complications associated with overuse of antibiotics. In addition, having strong local ID champions, who already had established relationships with electrophysiology providers was advantageous, as this helped to bridge the external facilitators with local site providers; this was critical for identifying barriers, ensuring that implementation of the intervention went more smoothly, building trust across stakeholders, and ensuring that engagement and communication with electrophysiology providers were ongoing. Furthermore, the ID champions were able to leverage leadership buy-in from department heads, such as the chief of cardiology, and antimicrobial stewardship program leaders, which helped to show electrophysiology providers that leadership strongly supported the intervention and practice change (i-PARIHS construct *Context-Leadership support*).

#### External facilitation

The study team, as external facilitators, offered sites with guidance and support needed to leverage practice change, including brainstorming implementation strategies and mechanisms for resolving barriers that arose during the facilitated implementation period. Having the study team present at the educational sessions provided additional expertise where electrophysiology providers and ID champions could ask specific questions about the evidence supporting discontinuation, patient outcomes, and information shown in the dashboard data. Implementing and adapting the dashboard based on local context helped to support sites’ surveillance and monitoring activities, particularly given the limited resources available for sites to dedicate their time to do this (i-PARIHS construct *Recipients—Time*). The study team had regularly scheduled meetings with ID champions to discuss progress and trends shown in the dashboard. In addition, the study team supported ID champions to collaboratively address challenges by brainstorming together and identifying strategies that would promote practice change and mitigate the barriers they were experiencing. Relationship building occurred through connecting electrophysiology providers with other peers to discuss concerns and hear about their experiences.

#### Providers’ readiness to collectively make the practice change together

Across all three sites, electrophysiology providers took steps toward making changes in their practice. A strong driver in taking these steps was their readiness to collectively change practice together (i-PARIHS construct *Recipients–Personal attributes, Context-Culture*). The electrophysiology providers had discussions about the evidence and the dashboard data and viewed that making the practice change would be aligned with evidence-based decision-making. Electrophysiology providers emphasized that the strength in the evidence acted as a “catalyst” to change their practice. However, electrophysiology providers at each site changed their practice at different increments—some occurred collectively after one discussion; others occurred after several discussions. While some electrophysiology providers had more initial skepticism about changing their practice, collective support among their peers helped in adopting these new practices and knowing that they were not the only ones to change their antimicrobial prophylaxis prescribing practice.

However, there were some challenges experienced among electrophysiology providers in getting them to make collective practice changes. Some providers who practiced both inside and outside of the VA were more resistant to change. In addition, practices external to the VA often influenced practices internal to the VA. Furthermore, while electrophysiology providers desired to make the practice change, existing order sets needed to be updated to help sustain the practice.

## Discussion

In this multicenter Hybrid III study, we found that an I/D bundle intervention that incorporates audit-and-feedback about harms of guideline-discordant care with infection surveillance reports to monitor and identify any unintended consequences of the practice change coupled with education, blended facilitation, and use of local trusted thought leaders was an effective strategy for reducing ineffective antimicrobial use practices in the cardiac electrophysiology laboratory. Practice improvements were not associated with any increases in CIED infection and there was a long-term decrease in antimicrobial-associated adverse events, supporting the clinical basis of the intervention and the potential for de-implementation efforts to improve patient-level outcomes and reduce unnecessary costs. Importantly, positive practice changes and improvements in clinical outcomes achieved during the facilitated implementation were sustained for more than a year following the active implementation phase, suggesting that the I/D bundle intervention led to long-term improvements in use of low-value care and that the practice improvement was associated with improvements in clinical outcomes.

The theoretical underpinning of this work was that information silos contributed to maintenance of the ineffective practice of prolonged prophylaxis; specifically, that providers administer prolonged antimicrobials to reduce the incidence of harms they see clinically and are therefore aware of (20). These same providers do not receive information about the downsides of this approach, creating a positive feedback loop that re-enforces the low-value practice. We hypothesized that breaking this feedback loop with better, more comprehensive feedback could improve care through a learning and unlearning process. The success of our study suggests that similar comprehensive informational strategies could be applied in other areas in which ineffective care is driven by unbalanced information about harms and benefits of different approaches. Of note, our deimplementation approach focused on giving providers feedback about *direct* harms to the patients they are caring for; we did not focus on population-level or future harms, such as the specter of antimicrobial resistance. While antimicrobial resistance is a critical public health threat, it is one that occurs primarily on a population level whereas provider decision making occurs at the patient level. This alternative strategy—focusing on specific harms in specific patients—may explain why this informational intervention was effective where others have demonstrated minimal or no impact of audit-feedback strategies.

A major benefit of our work is that the building blocks of the informational dashboard are inherently designed to be portable and adaptable to address antimicrobial overuse in other settings (Figure 4). Although clinical guidelines about pre- and post-procedural antimicrobial use vary by clinical specialty and electronic algorithms measuring compliance need adaptation prior to use in other areas, antimicrobial harms electronic measurement tools can be directly applied without additional change. In addition, over the course of prospective deployment, we refined and improved our AI-facilitated CIED infection algorithm, highlighting feasibility of iterative refinement of informatics tools for patient safety monitoring activities (28). Dashboard elements represent building blocks of a true learning health system, in which validated algorithms can be mixed and matched in different ways to support an ongoing quality improvement cycle with electronic data.

**Figure 4:**
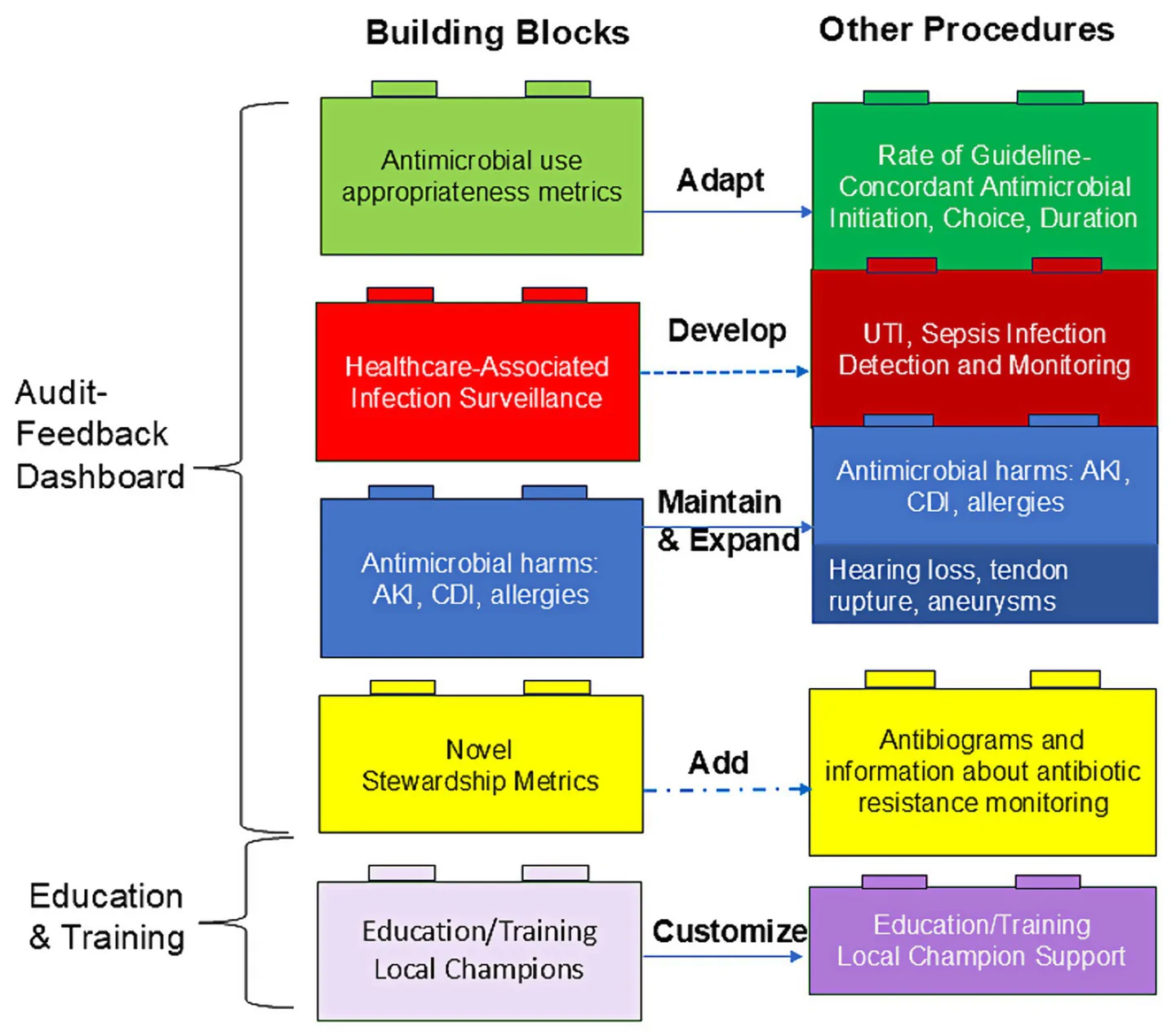
Building blocks for a learning health system approach to audit-feedback interventions.

At one of the three sites, many of the electrophysiologists provided care at non-VA sites, with other standards of care that dictated prolonged prophylaxis, potentially contributing to lack of receptiveness to practice change and limited uptake of the deimplementation effort. A major lesson of this de-implementation study was that practices that are imported into the VA from other settings are very difficult to change using VA-based audit-and-feedback metrics; the de-implementation bundle was least effective when non-VA frames of reference were used for benchmarking rather than the information provided through the dashboard. A second major lesson is that audit-and-feedback can be an opportunity to review existing practices and factors associated with long-term sustainment; this has implications not just for the specific question of discontinuing inappropriate antimicrobial use but also for other interventions that are on autopilot after integration into the EHR. For example, the practice was maintained at one site primarily due to an old order set that had not been removed from the system.

We also found that engagement throughout the entire healthcare support team was critical for achieving success; targeting electrophysiologists only was ineffective for achieving practice change. This required mapping of processes of care and identification of all individuals primarily involved in the post-procedural care of patients undergoing CIED procedures and education of all stakeholders of the change and the rational for the change. Prior to the nursing education session, nurses paged physicians requesting orders prior to discharge. This encouraged ongoing practice to reduce burden of pages and to save time, not because the practice was evidence-based. After physician education and agreement to change, ongoing education of nursing was necessary at one site to provide reassurance that the de-implementation was both deliberate and safe, based on medical evidence. Leadership support in the form of endorsement from cardiology leadership was also important to place emphasis on the importance of the change and to provide a catalyst for de-implementation efforts. Effective de-implementation requires identification of each of the factors that encourage continuation of long-standing practice and active efforts and strategies to address each one.

This study has several limitations. First, it was conducted in the closed VA healthcare system. This meant that many of the providers were primarily employed by the VA and thus motivated by VA-wide benchmarking strategies. As suggested by limited impact at one of the study sites with high proportions of providers importing practice patterns from other systems, similar efforts may be less effective outside of the VA if facility-level comparisons are not available to address legal liability concerns. Second, the intervention deliberately targeted facilities with high rates of non-compliance with current guidance. This meant that there was a larger potential for improvement than at facilities with lower rates of non-compliance but with non-compliance none-the-less present. However, as part of the bundle, we leveraged differences in practice to highlight to providers the safety of the intervention and thus this approach was inherent to the audit-feedback with benchmarking strategy used as part of the I/D bundle. Demonstration of similar outcomes at sites that de-implemented prolonged use was an important strategy for addressing provider liability concerns about current standards of care should a CIED infection occur. Findings would necessarily be less impactful at facilities with higher baseline rates of guideline compliance and lower rates of prolonged antibiotic use. Third, our *a priori* data analysis plan included comparing the sites pre/post implementation rather than using a matched controlled design. We opted to use a matched controlled design to improve our ability to account for temporal trends among similar VA facilities. However, the original analysis plan would have demonstrated similar findings, with substantial and sustained improvements in two of the three intervention sites. Fourth, we used a non-randomized parallel group design with matched controls rather than a randomized controlled trial of sites with high rates of non-compliance. The use of a parallel group trial is different from our initial stepped-wedge design but is a higher-quality study design and, although not a randomized controlled trial, is aligned with the inherently quality improvement aspects of this proposal. Fifth, relative to the initial study protocol, we added additional antimicrobial harm metrics, specifically, the inclusion of antimicrobial allergies. We also analyzed a composite outcome of antimicrobial harms (AKI, allergic events, *C. difficile* infections). Although the allergic event metric was not included in the initial study plan, we believe it improved, rather than detracted from the work.

## Conclusion

In this matched, prospective quasi-experimental cohort study, we found that an I/D bundle of strategies was effective for reducing low-value care. The I/D bundle can be adapted and applied in other settings to reduce low-value practice and improve quality and outcomes.

## Data Availability

The datasets presented in this article are not readily available because this study was conducted in the VA. VA researchers with relevant access can contact the corresponding author with reasonable requests aligned with VA data use agreements and protections. Materials to support implementation efforts are included as Supplementary files. Requests to access the datasets should be directed to westyn.branch-elliman@va.gov.
