# Supplementary material for: Balanced informational feedback as part of an implementation/deimplementation bundle to support deimplementation efforts in antimicrobial stewardship: results from a mixed methods, hybrid type III implementation/effectiveness trial
Source: Front Public Health. 2026 Sep 23;14:1893058. doi: 10.3389/fpubh.2026.1893058 (PMC13642756; doi:10.3389/fpubh.2026.1893058)
Supplement: Supplementary file 1 [file Data_Sheet_1.PDF]

PROMOTING DE-IMPLEMENTATION OF INAPPROPRIATE ANTIMICROBIAL USE FOLLOWING CARDIAC DEVICE PROCEDURES

Westyn Branch-Elliman, MD, MMSc

VA Boston Healthcare System

Harvard Medical School

ONE VETERAN'S STORY

- 67-year-old male presents to clinic to discuss ICD implantation
  - Reports falling out of a chair a few weeks ago; unclear if he just fell or lost consciousness
  - Reports occasional palpitations but denies CP, orthopnea, and edema currently
- May 10: BiV ICD placement given severely reduced EF
- May 11: discharged with a prescription of Keflex 500mg for the next 7 days to prevent CIED infection
- May 20: admitted back to the hospital with reports of 4 days of bloody diarrhea
- June 6: tested positive for C. diff; prescribed Metronidazole to treat the infection in colon, which doctors said was related to the Keflex taken to prevent CIED from getting infected
- Did not appear to be getting better so prescribed another antibiotic (oral Vancomycin)
- Continued to have diarrhea as well as severe and progressive abdominal pain
- Consulted with surgery to discuss possibility of removing colon to control infection
- Evaluated by surgery; determined that patient too sick for operation
- Admitted to ICU due to worsening symptoms (high fever, mental confusion)
- June 30: Passed away in ICU from sepsis due to C. difficile infection

ANOTHER VETERAN'S STORY

- Overweight 65-year-old Veteran with a history of heart palpitations, who has recently been experiencing high fatigue
- January 2: Presents to clinic to discuss ICD implantation
  - In the past, patient has been skeptical of an ICD and does not think one is needed
  - At this checkup appointment, agrees to do the procedure.
- January 18: successfully undergoes the ICD procedure without any complications
  - Received the appropriate antibiotics before the procedure.
- January 25 (prior to discharge): briefed on how to avoid infections and properly care for wound
  - Not given any post-procedural antibiotics.
- January 26 (check in telephone call): reports no issues and expresses feeling "very good" and going on "long walks with family and enjoying a more active lifestyle"

WHEN ARE ANTIBIOTICS HELPFUL FOR PREVENTING PROCEDURE-RELATED INFECTIONS?

PRE-INCISION ANTIMICROBIAL PROPHYLAXIS: EVIDENCE-BASED GUIDELINES AND POLICY

Evidence-based and guideline-recommended practice

Antibiotics within 1 hour window prior to skin incision	Discontinuation within 24 hours after skin incision

Due to the high quality of evidence supporting appropriate pre-incision prophylaxis and early discontinuation, measurement and reporting of antimicrobial use measures were included in the Surgical Care Improvement Program (SCIP)

**INF-1**: Prophylactic antibiotic received within one hour prior to surgery	**INF-2**: Appropriate pre-procedure antibiotic selection	**INF-3**: Discontinuation of antibiotic within 24 hours of skin closure (48 hours for cardiac surgery)

Bratzer et al, The Joint Commission.
Bratzler et al. American Journal of Health-System Pharmacy. Feb 2013.
Stone PA et al. Annals of Surgery. Sept 2015.
Courtney PM. Clin Orthop Relat Res. July 2015.

CIED PROCEDURE SPECIFIC DATA

- **PRE-OPERATIVE ANTIMICROBIALS:**
- Randomized Controlled Trial (RCT)
- Planned for N=1000 patients
- Stopped early due to lower event rate in prophylaxis arm
  - Total of 314 patients received cefazolin, whereas 335 patients received placebo
  - Safety committee interrupted study after 26.5 months due to a significant difference in the infection rate between the 2 groups
  - Primary end point of infection reached in 2 (0.64%) patients in the cefazolin group and 11 (3.28%) patients in placebo group (*P*=0.016)

- **POST-OPERATIVE ANTIMICROBIALS:**
- Open-label RCT
- Device procedures performed in 28 centers in 19,603 patients, of whom 12,842 were high risk
  - Infection occurred in 99 patients (1.03%) receiving conventional treatment, and in 78 (0.78%) receiving incremental treatment (odds ratio: 0.77; 95% confidence interval: 0.56 to 1.05; p = 0.10)
  - In high-risk patients, hospitalization for infection occurred in 77 patients (1.23%) receiving conventional antibiotics and in 66 (1.01%) receiving incremental antibiotics (odds ratio: 0.82; 95% confidence interval: 0.59 to 1.15; p = 0.26)
  - Subgroup analysis did not identify relevant patient or site characteristics with significant benefit from incremental therapy
- Conclusion: No benefit to post-procedural antimicrobials in cardiac device implantation procedures

DeOliviera et al, Circulation: Arrythmia and Electrophysiology. 2009

ANTIMICROBIAL ADVERSE EVENTS

Antimicrobials are a leading cause of emergency room visits for adverse drug events

Risks of antimicrobials are diverse, and include acute kidney injury, *C. difficile* infection, rash/anaphylaxis, and antimicrobial resistance

Little is known about the impact of type and duration of antimicrobial prescription on the risk of common adverse events with immediate consequences

Crabtree et al. Ann Thor Surg. 2007.
Shehab et al. JAMA. 2016.
Filippone et al. Clin Pharm Ther 2017.
Courtney PM. Clin Orthop Relat Res. July 2015.

DAILY RISK AND NUMBER NEEDED TO HARM

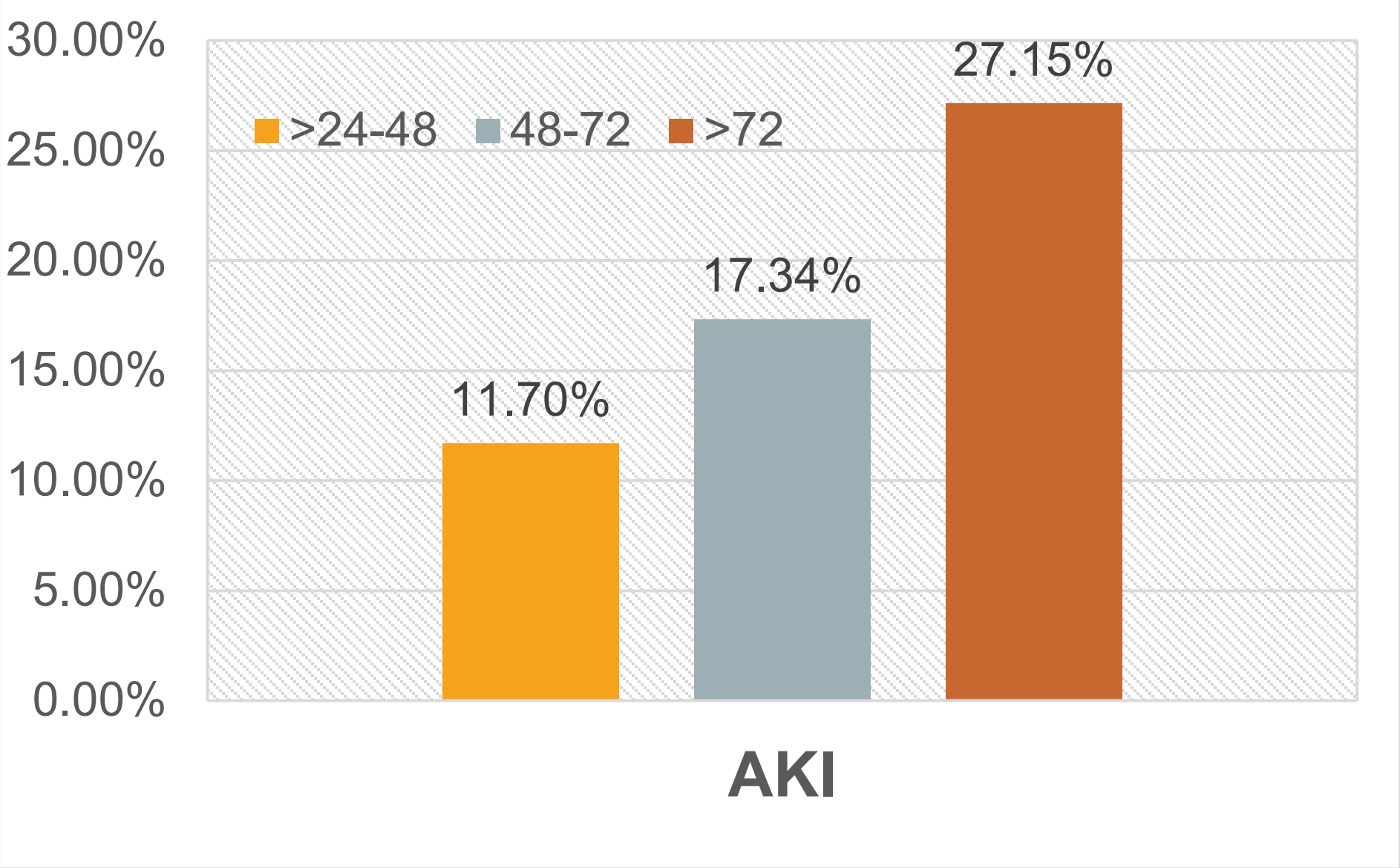

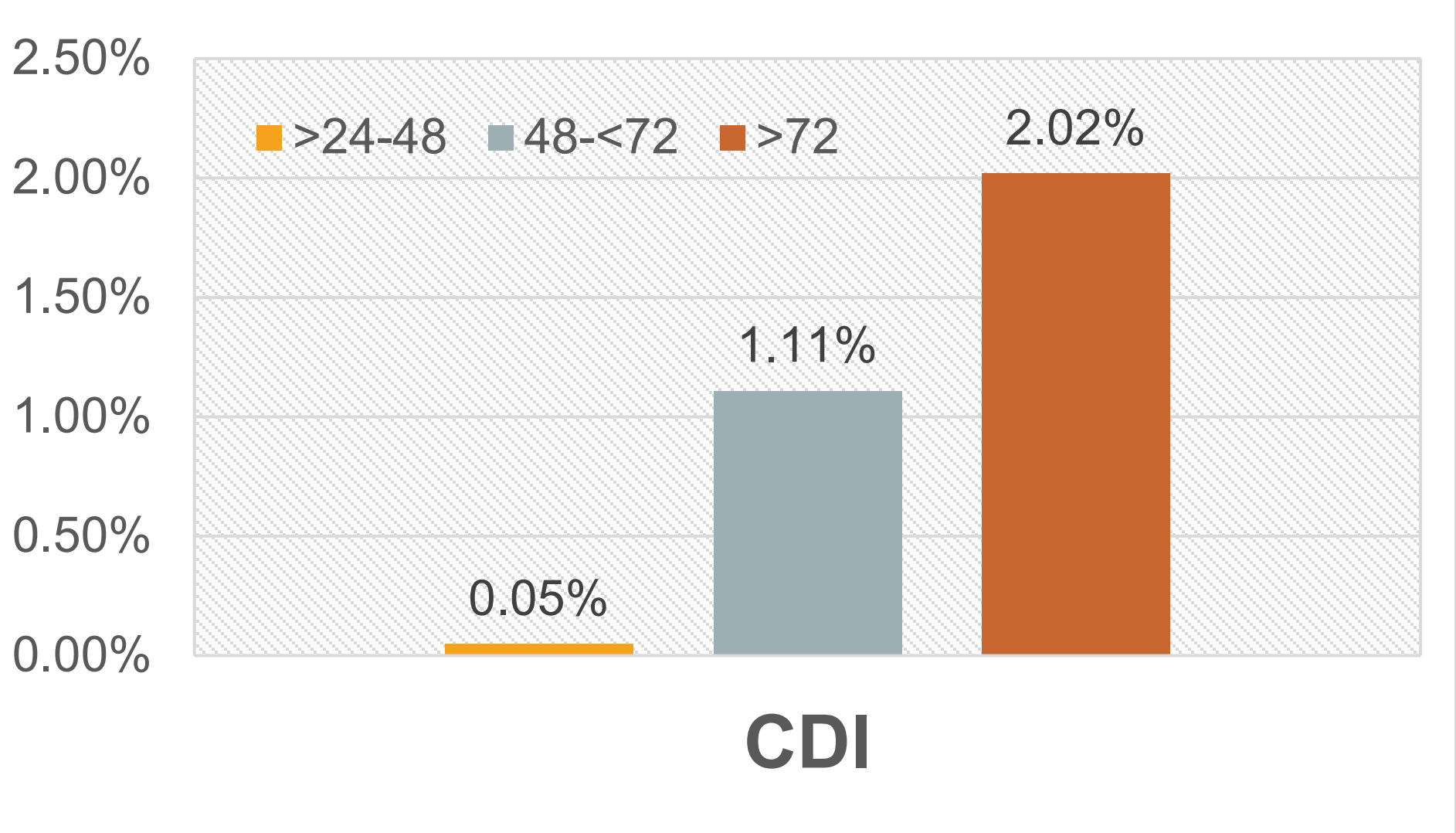

NNH	AKI	CDI
>24-48	8.55	2000
>48-72	5.77	90.09
>72	3.68	49.50

*AKI= Acute kidney injury. CDI= *Clostridium difficile* infection. NNH= Number needed to harm. Results are unadjusted and pooled for all surgical types.

DAILY ADJUSTED ODDS OF ADVERSE OUTCOMES

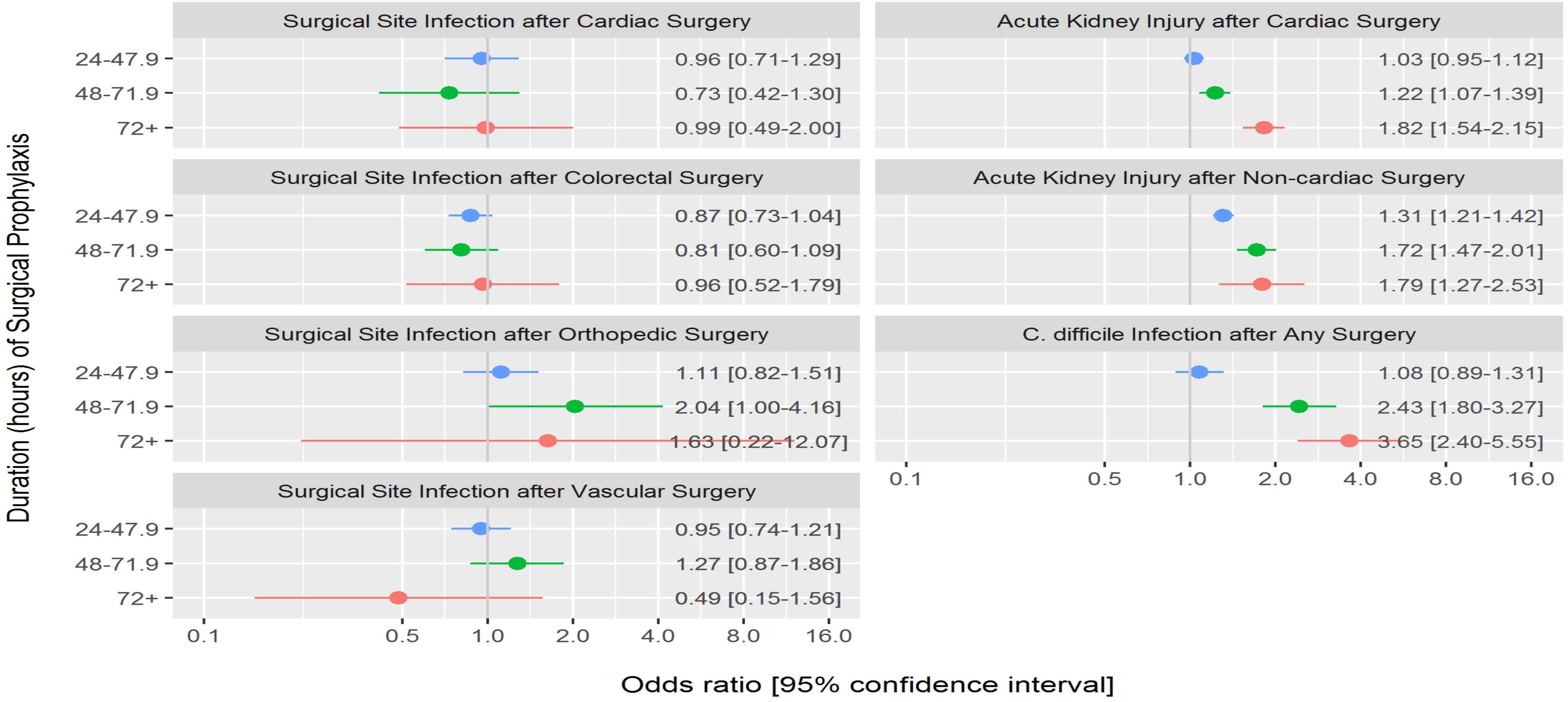

IMPACT OF COMBINATION REGIMENS ON AKI

Variable	Adjusted RR (95%CI)	Number Needed to Harm/Treat*
Any AKI	1.20 (1.15-1.25)	18.69
AKI Stage 1	1.20 (1.15, 1.26)	20.79
AKI Stage 2	1.30 (1.12, 1.52)	87.72
AKI Stage 3	1.47 (1.19-1.82)	116.28
SSI	0.62 (0.48, 0.81)	166.67*

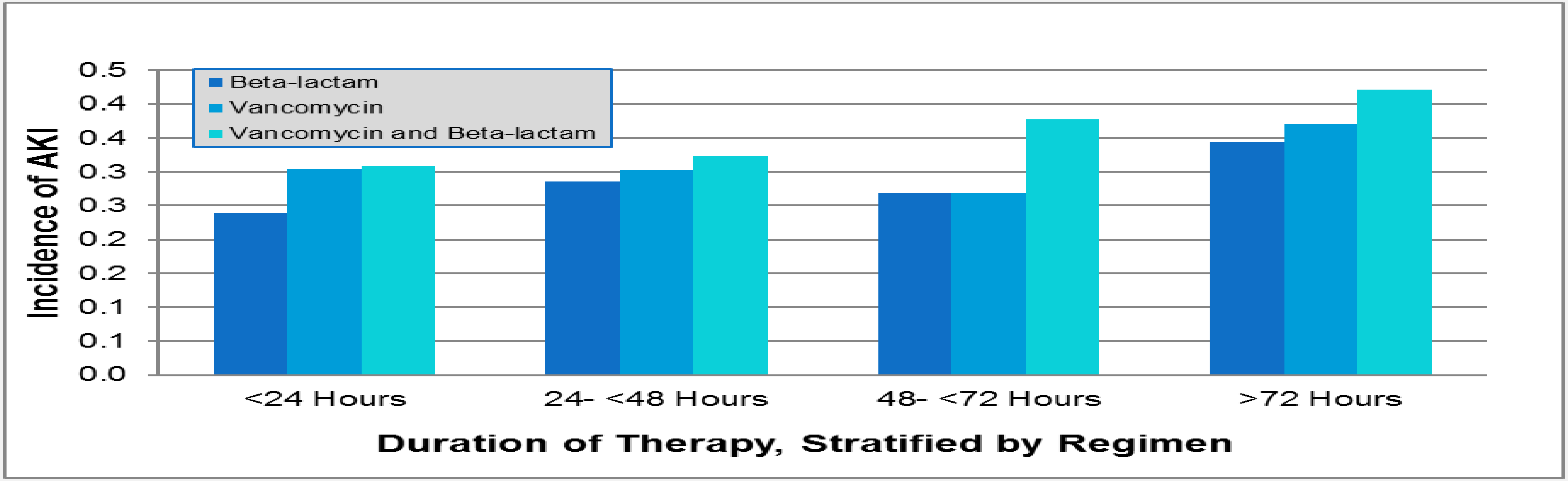

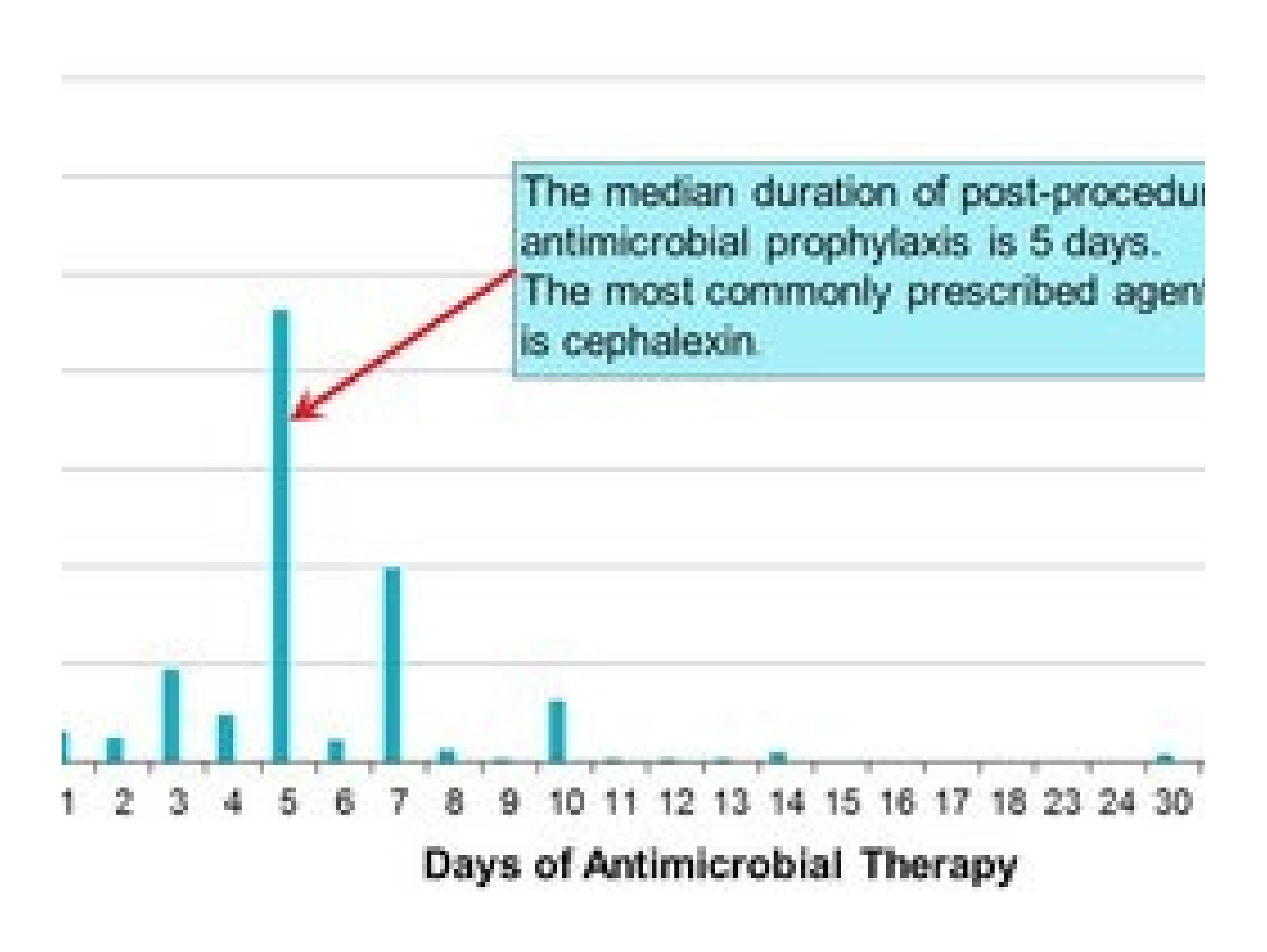

DIFFUSION OF SCIP METRICS TO PROCEDURAL SPECIALTIES (EP) VERY LIMITED

HIGH RATES OF POST-PROCEDURE ANTIMICROBIAL USE FOLLOWING CARDIAC DEVICE PROCEDURES

Source: Branch-Elliman et al, ICHE, 2016

DO THE EXTRA DAYS OF ANTIBIOTICS REALLY MATTER?

A. Relationship Between Postprocedure Antimicrobial Exposure and *C. difficile* Infection (Cox Proportional Hazards Model)						
			Unadjusted		Adjusted[a]	
Total Cases	No. of Events	Exposure	HR (95% CI)	*P* Value	HR (95% CI)	*P* Value
6,497	27	Prolonged antibiotics (>24 h)	2.72 (1.46-5.07)	.002	2.90 (1.54-5.46)	.001
		Total days of antibiotics[b]	1.04 (1.02-1.05)	<.001	1.03 (1.02-1.05)	<.001

B. Relationship Between Postprocedure Antimicrobial Exposure and Acute Kidney Injury (GEE Model)						
			Unadjusted		Adjusted[c]	
Total Cases	No. of Events	Exposure	OR (95% CI)	*P* Value	OR (95% CI)	*P* Value
2,797	222	Prolonged antibiotics (>24 h)	1.24 (0.80-1.90)	0.336	1.26 (0.84-1.91)	.267
		Total days of antibiotics	1.01 (0.98-1.04)	0.475	1.01 (0.98-1.04)	.50
		No. of antibiotics - 1 (vs none)	0.92 (0.61-1.38)	0.683	0.93 (0.63-1.38)	.718
		No. of antibiotics - 2 or more (vs none)	4.01 (2.48-6.47)	<.001	4.16 (2.50-6.90)	<.001
		Vancomycin+other antibiotic	7.87 (5.37-11.54)	<.001	8.41 (5.53-12.79)	<.001

EVERY DAY MATTERS: THE DURATION-DEPENDENT RELATIONSHIP BETWEEN POST-OPERATIVE ANTIBIOTIC USE AND POST-OPERATIVE OUTCOMES

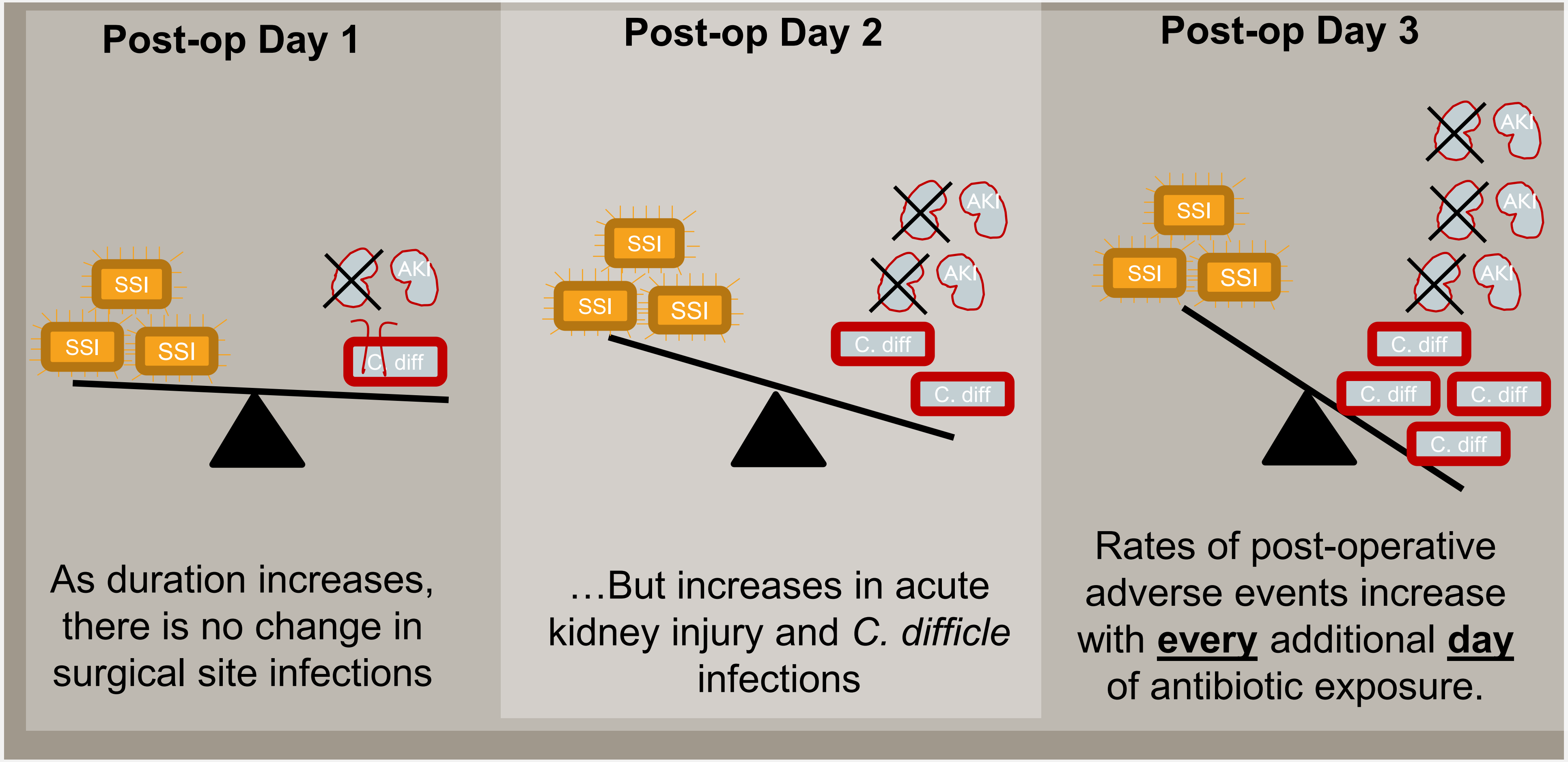

JAMA Surgery

PRE-Procedure Antibiotics Reduce Infections and IMPROVE Outcomes

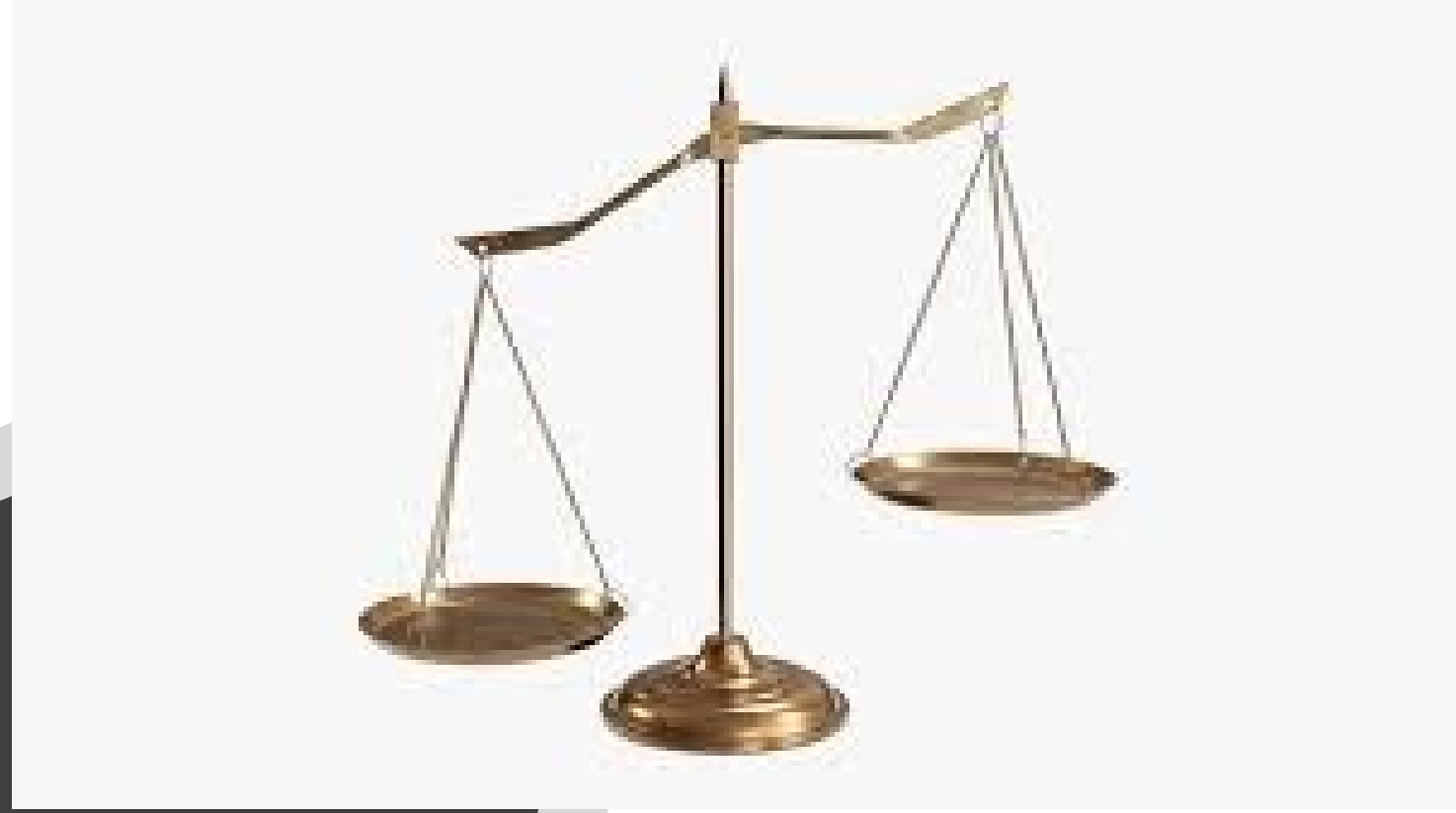

Peri-operative antibiotics: A double-edged sword

POST-PROCEDURE Antibiotics DO NOT Reduce Infections but they DO INCREASE HARM

EVIDENCE-BASED PRACTICES FOR PREVENTING PERI-OPERATIVE AND PERI-PROCEDURAL INFECTIONS

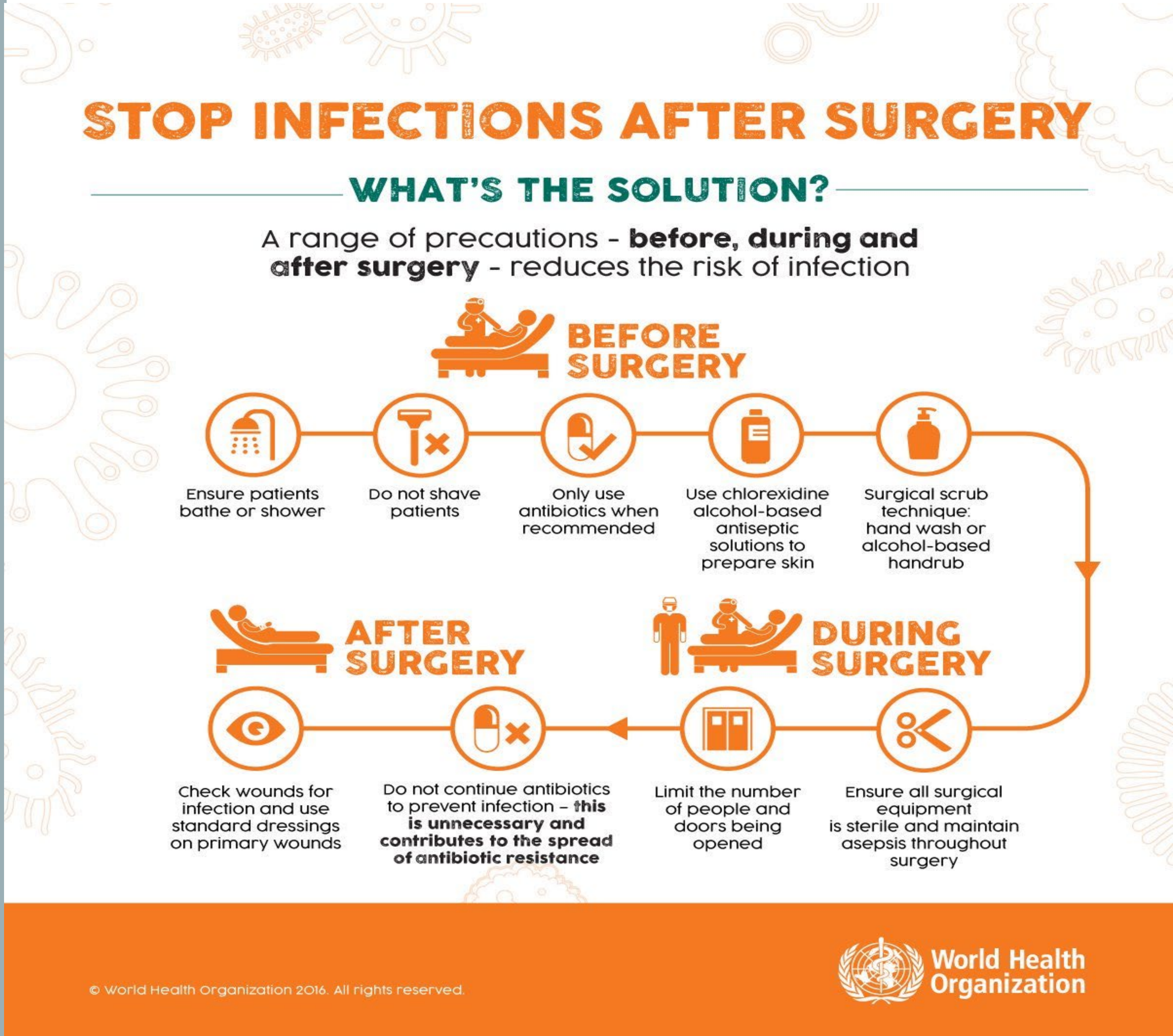

WHAT STRATEGIES ARE EFFECTIVE FOR REDUCING PROCEDURE-RELATED CIED INFECTIONS?

	• Continue or interrupt temporarily if possible	Reduce risk for pocket haematoma
• Antiplatelets?	• Discontinue 5-10 d prior surgery (particularly P2Y12 inhibitors) & avoid DAPT if possible	
• Steroid treatment?	• Is withdrawal or dose reduction possible?	
• Is procedure complex/expected to be lengthy?	• Consider experienced operator and/or supervisor to shorten procedure time or consider referral to experienced operator/high volume centre	
• CIED replacement?<br>• Upgrade to more complex CIED?<br>• Early re-intervention?	• Re-evaluate indication for replacement/upgrade.<br>• Does the benefit of device implantation outweigh the risks?<br>• Consider alternative approach to transvenous system.<br>• Postpone procedure if possible	
• Presence of many leads and/or abandoned leads?	• Consider extraction on individual basis	
• High-risk patient for infection?	• Consider LPM, S-ICDs or epicardial system if appropriate.<br>• Reconsider indication for device implant<br>• Consider experienced operator or refer to high volume centre if complex procedure	
• *S. aureus* colonization	• -Consider nasal swabs and nasal treatment with mupirocin and chlorhexidine skin washing in selected patients	
• Is i.v. antibiotic therapy given?	• i.v. flucloxacillin (1–2 g) or cefazolin (1–2 g) within 1 h prior to surgery	
• Is procedure scheduled as 'out-of-hours' procedure?	• Postpone procedure to be performed during office hours	
Intra-operative actions		
• High-risk patient for infection?	• Consider antibiotic-impregnated mesh envelope (minocycline/rifampicin)<br>• Ensure short procedure times and low complication rate by selecting experienced	

EVIDENCE-BASED PREVENTION INTERVENTIONS

Table 2 Recommended actions for prevention of device infections according to EHRA consensus document

Consensus statement	Scientific evidence coding
Pre-procedural actions	
Confirm indication for CIED	E
Delay CIED implantation in patients with infection	E
Avoid temporary transvenous pacing and central venous lines, which should ideally be removed prior to introducing new hardware, whenever possible	O, M
Measures to avoid pocket haematoma are recommended (avoid heparin bridging, discontinue antiplatelets if possible)	R
Periprocedural use of therapeutic Low-molecular-weight-heparin	R, M, O
Perform the CIED procedure in an operating room/suite with complete sterile environment as required for other surgical implant procedures.	E
Procedure should be performed or supervised by an operator with sufficient training and experience	O
• Operators with ∼<100 CIED procedures experience should work under close supervision of more experienced operators	O, E
• An annual minimum operator volume of ∼50 CIED procedures is recommended for all operators	
Topical *S. aureus* decolonization may be performed	E
Pre-procedural skin wash may be performed	E
Hair removal with electric clippers (not razors) is recommended	O
Antibiotic prophylaxis is recommended within 1 h of incision for cefazolin and flucloxacilline, within 90–120 min for vancomycin	R, M
A continuous surveillance program of infection rates and associated microbiology should be set-up at the level of each implanting centre	E
Intra-procedural actions	
Surgical preparation with alcoholic chlorhexidine should be used rather than povidone-iodine	R
Allow sufficient time for the antiseptic preparation to dry	E
Adhesive iodophor-impregnated incise drapes may be used	E
Perform the procedure with adequate surgical technique—minimize tissue damage, haemostasis, adequate wound closure	E
Antibiotic envelope in high-risk situations is recommended*	R
If the operator performs the prepping and draping, glove change/re-scrub or remove outer glove of a double-glove before incision	E
Using local instillation of antiseptic and antibiotics in the pocket	R, E
Use of braided sutures for final skin closure	E
Post-procedural actions	
Use of post-operative antibiotic therapy	R
Adequate dressing for 2–10 days is recommended	E
Patient instructions on wound care should be provided	E
Delay or reconsider indication for re-intervention if possible	E
Haematoma drainage or evacuation (unless tense, wound dehiscence is present or pain is severe)	O

Modified table from EHRA international consensus document on how to prevent, diagnose, and treat CIED infections.[20]
EHRA Statement classes; turquoise = recommended/indicated or 'should do this'; red = may be used or recommended; and green = should not be used or recommended.
EHRA ROME coding: R, randomized trials; O, observational studies; M, meta-analysis; E, expert opinion.
CIED, cardiac implantable electrical devices; LPM, leadless pacemaker; S-ICD, subcutaneous implantable defibrillator; *S. aureus*, *Staphylococcus aureus*.
*As defined in the WRAP-IT study population (ref 74) (patients undergoing pocket or lead revision, generator replacement, system upgrade, or an initial CRT-D implantation) and patients with other high risk factors, considering also the local incidence of CIED infections.

Blomstrom-Lundqvist. Europace. 2021

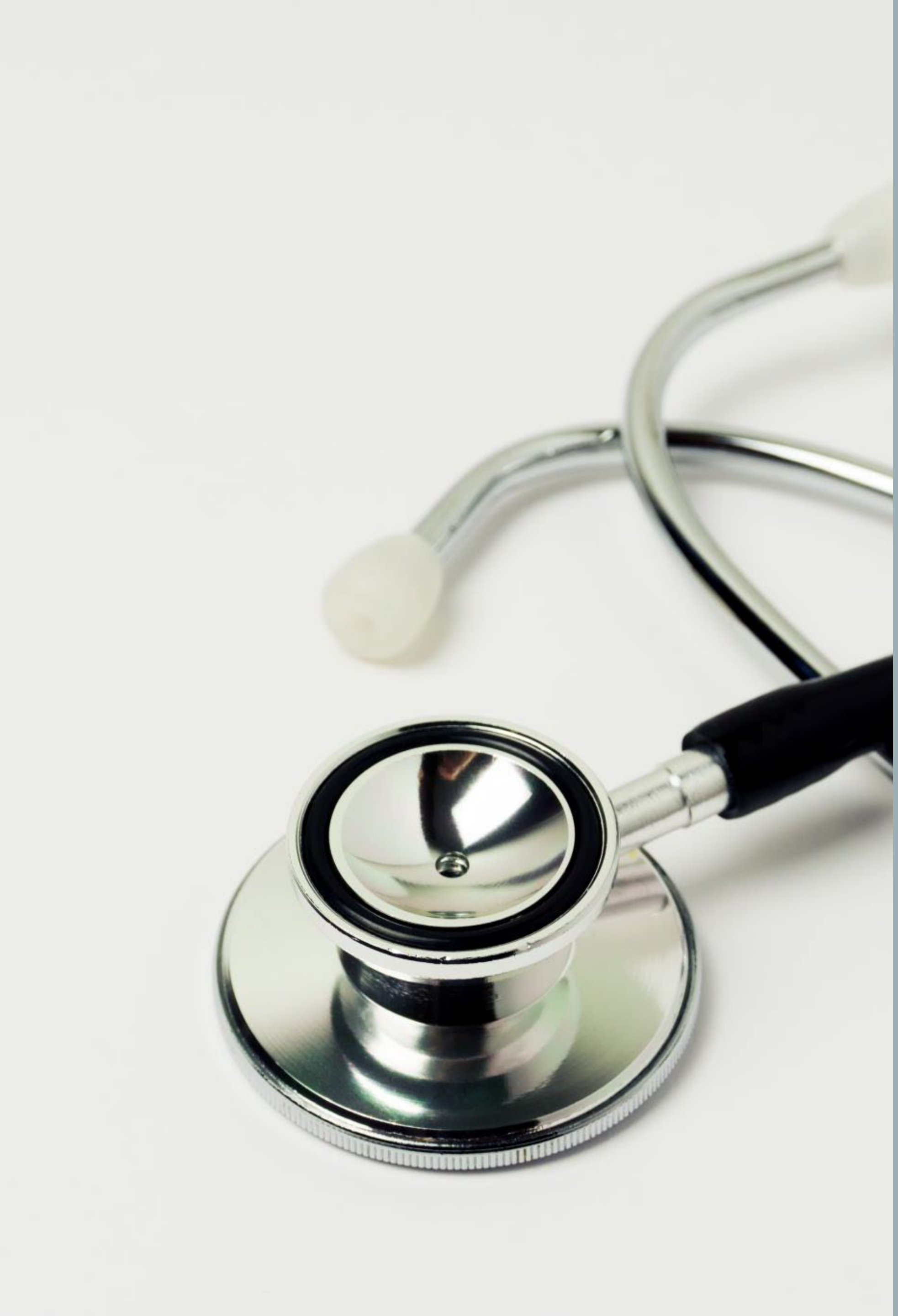

DOES THIS APPLY TO VA PATIENTS?

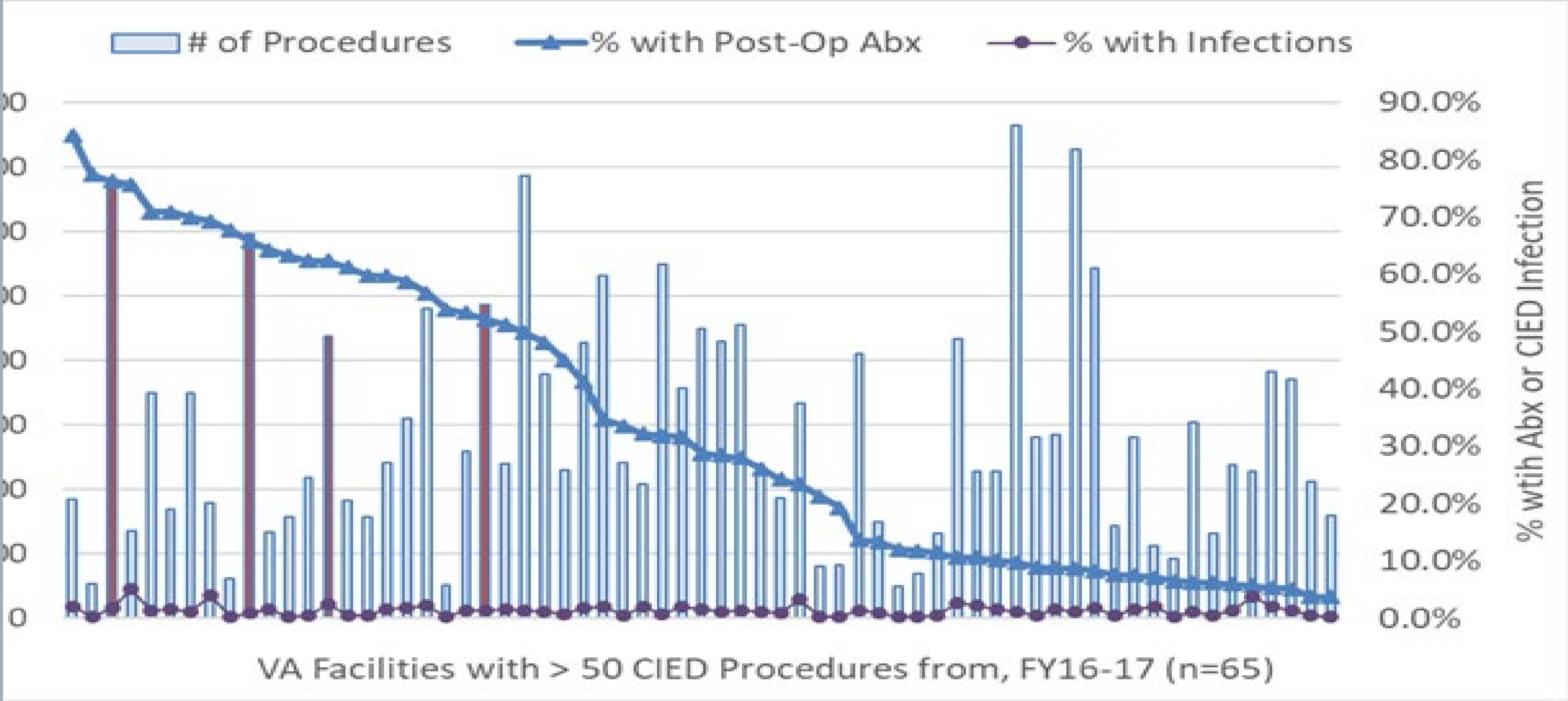

SUBSTANTIAL VARIATION IN PRACTICE ACROSS THE VA, BUT NO CORRELATION BETWEEN POST-PROCEDURE USE AND CIED INFECTIONS

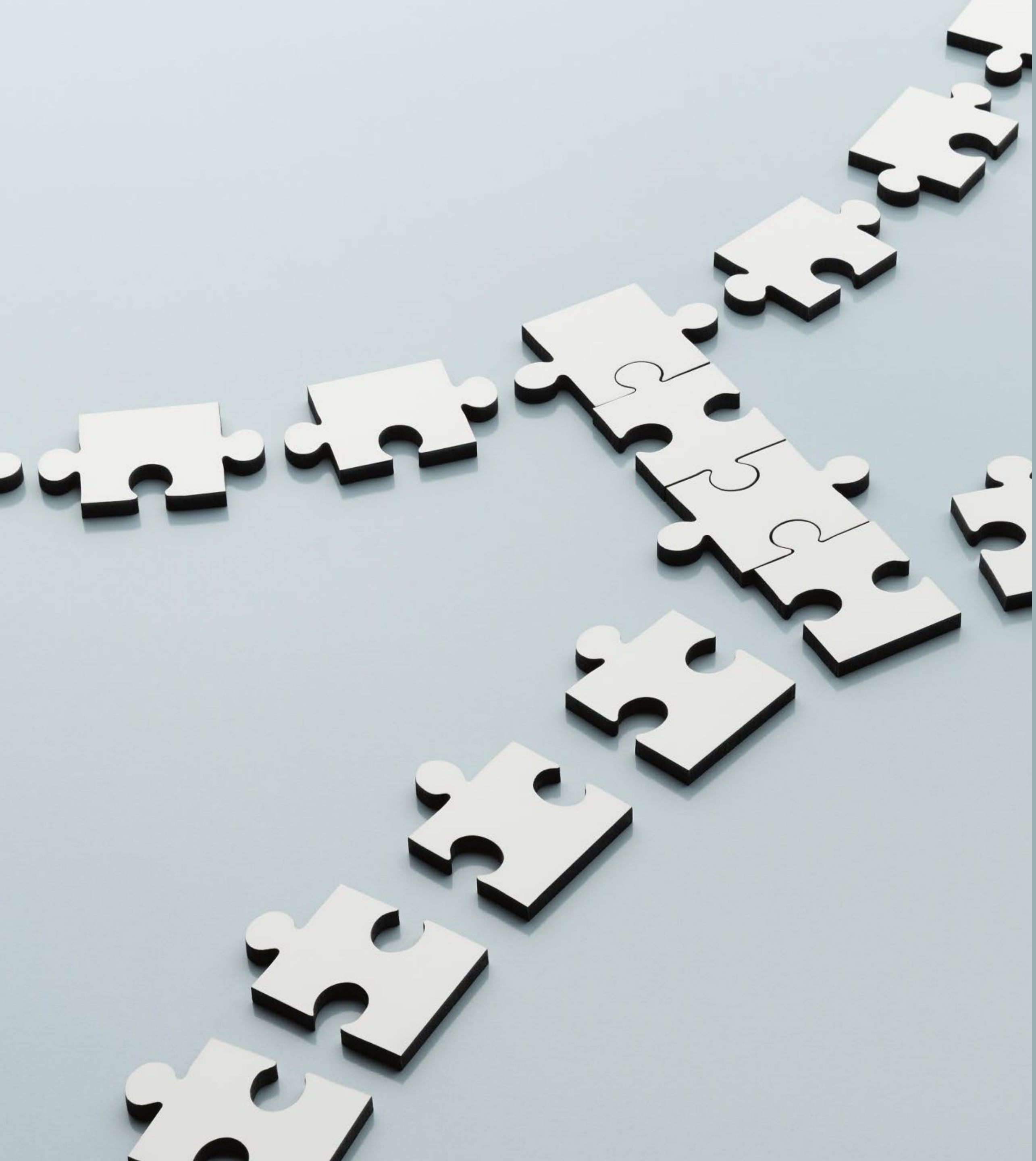

PUTTING THE PIECES TOGETHER

WHAT TO CONSIDER

Do I Need to Give Antibiotics?

YES

- **The patient has a proven bacterial infection** (i.e. a known pathogen, cultured at a significant level, that is likely to be the cause of the clinical presentation)
- **The patient's presentation is consistent with an immediately life-threatening infection** (e.g. suspected bacterial meningitis, meningococcal sepsis)
- **The patient is hypotensive due to suspected infection**

WATCH and WAIT*

- **The undifferentiated febrile patient who is not hypotensive**
- **The patient with a colonised catheter with a low virulence organism**
- **The patient with a ventilator-associated condition (VAC†)**

*Watch and Wait: Close monitoring of the patient in an high-dependency or intensive care setting for signs of deterioration whilst further investigations and attempts at source identification and control are carried out.

†VAC: An increase in the minimum PEEP during a 24-hour period of 3cm H_2O or an increase in the minimum FiO_2 during a 24 hour period of 20%, after a period of 48 hours of stable ventilator settings

1. Does my patient have an infection that requires antibiotics?
2. Have I ordered appropriate cultures before starting antibiotics? What empiric therapy should I initiate?
3. A day or more has passed. Can I stop antibiotics? Can I narrow therapy or change from IV to oral therapy?
4. What duration of antibiotic therapy is needed for my patient's diagnosis?

What Can Patients Do?

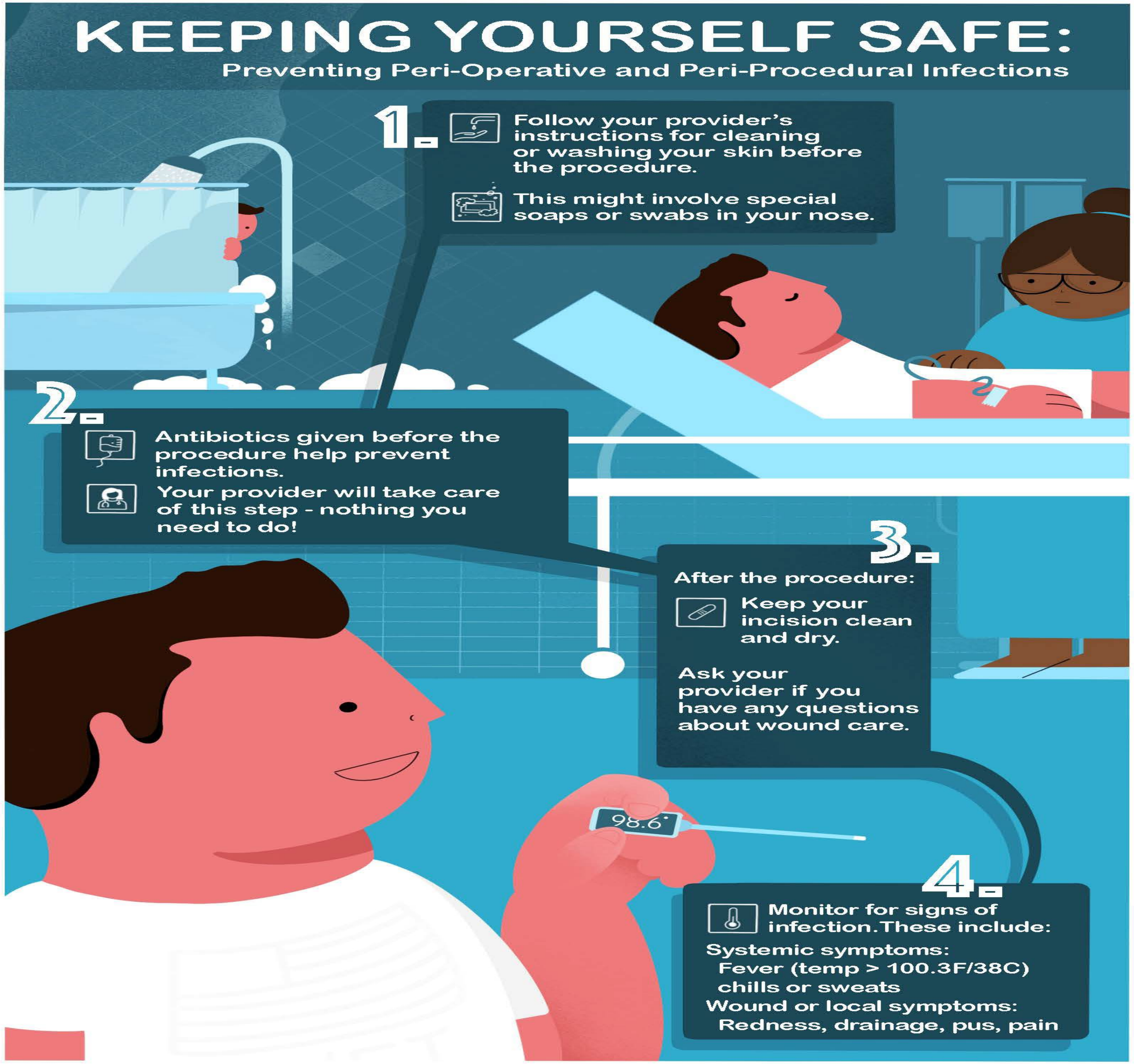

Post-Operative Infection – Signs to Look for (And Educate Patients about!)

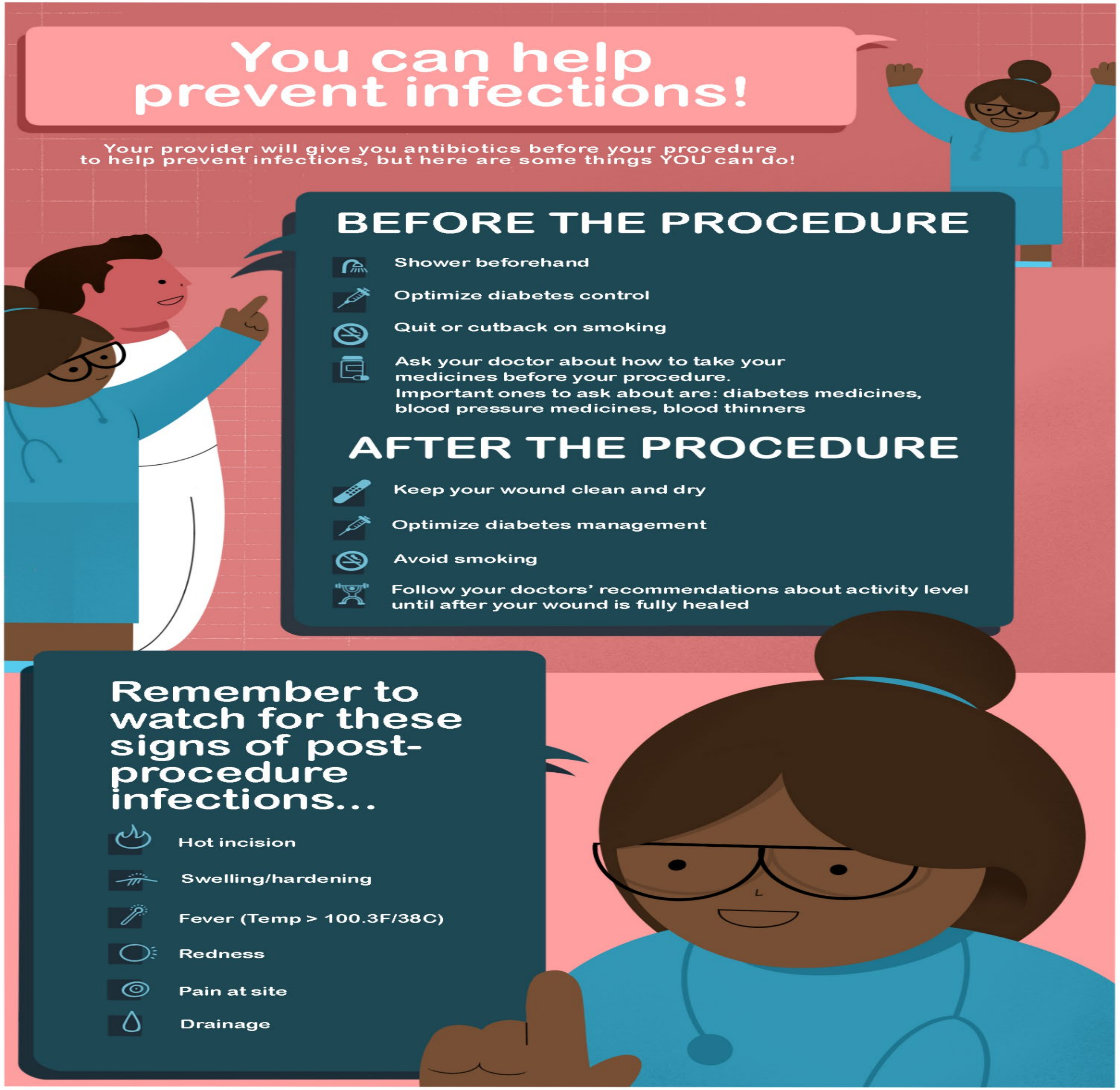

VERSION 1

DASHBOARD: ELEMENTS

Antimicrobial Use

Post-Procedure Antibiotic Use

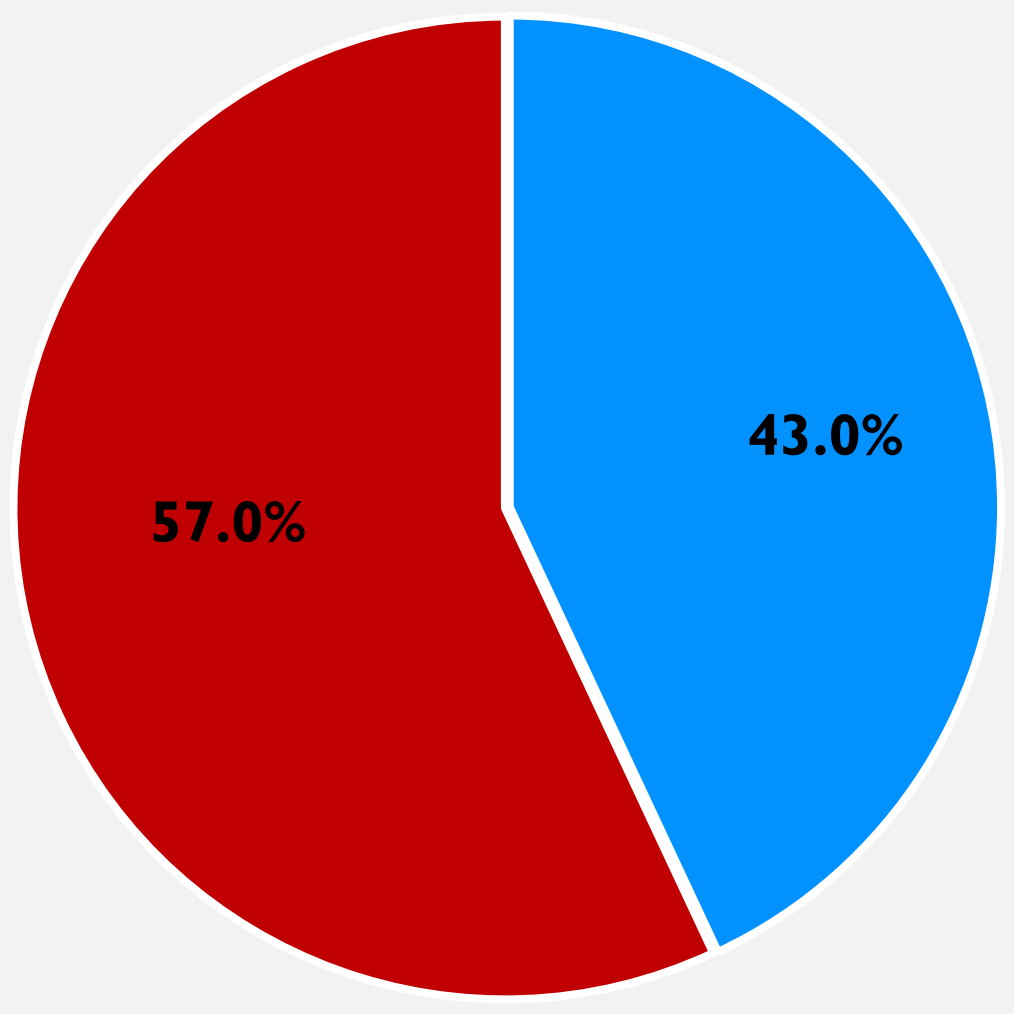

Clinical Outcomes

	Q3FY22	Q4FY22	Q1FY23
**Adverse Events**	**N (%)**	**N (%)**	**N (%)**
Cardiac Device Infection†	55 (3.0)	48 (2.7)	35 (2.1)
Acute Kidney Injury	75 (4.0)	58 (3.1)	63 (3.7)
C. difficile	0 (0.0)	2 (0.1)	3 (0.2)
Allergic Reaction	4 (0.2)	4 (0.2)	6 (0.3)
Total Procedures	*1,868*	*1,854*	*1,718*

† Algorithm-predicted infections are unconfirmed. Denominator for % is total number of patients.

VERSION 2

DASHBOARD: ELEMENTS

Antimicrobial Use

Post-Procedure Antibiotic Use

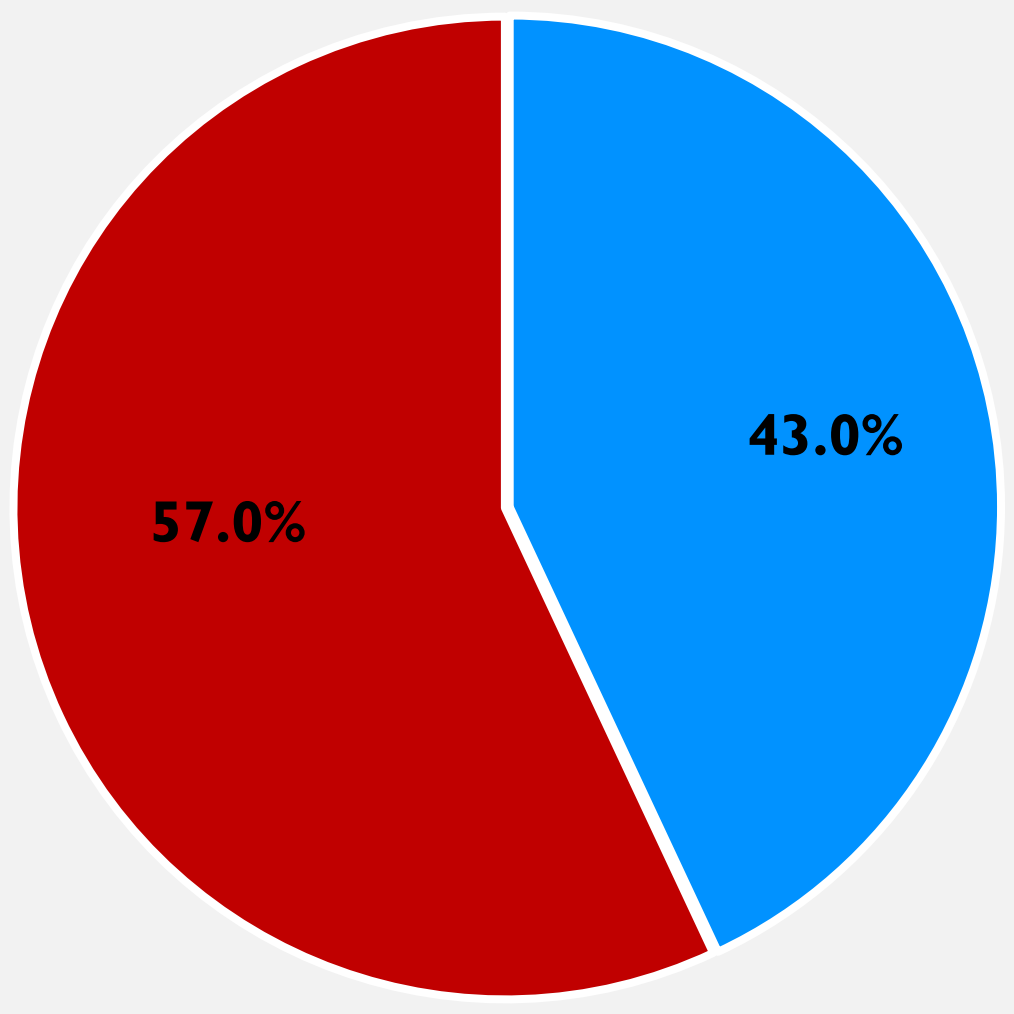

Clinical Outcomes

Clinical outcomes for CIED procedures between April2022 – Dec2022 (FY22Q3 – FY23Q1)	
**Adverse Events**	**N (%)**
Cardiac Device Infection†	138 (2.7)
Acute Kidney Injury	196 (3.6)
C. difficile	5 (0.1)
Allergic Reaction	14 (0.3)
Total Procedures	*5,440*

† Algorithm-predicted infections are unconfirmed. Denominator for % is total number of patients.

DASHBOARD DEMONSTRATION- POWER BI

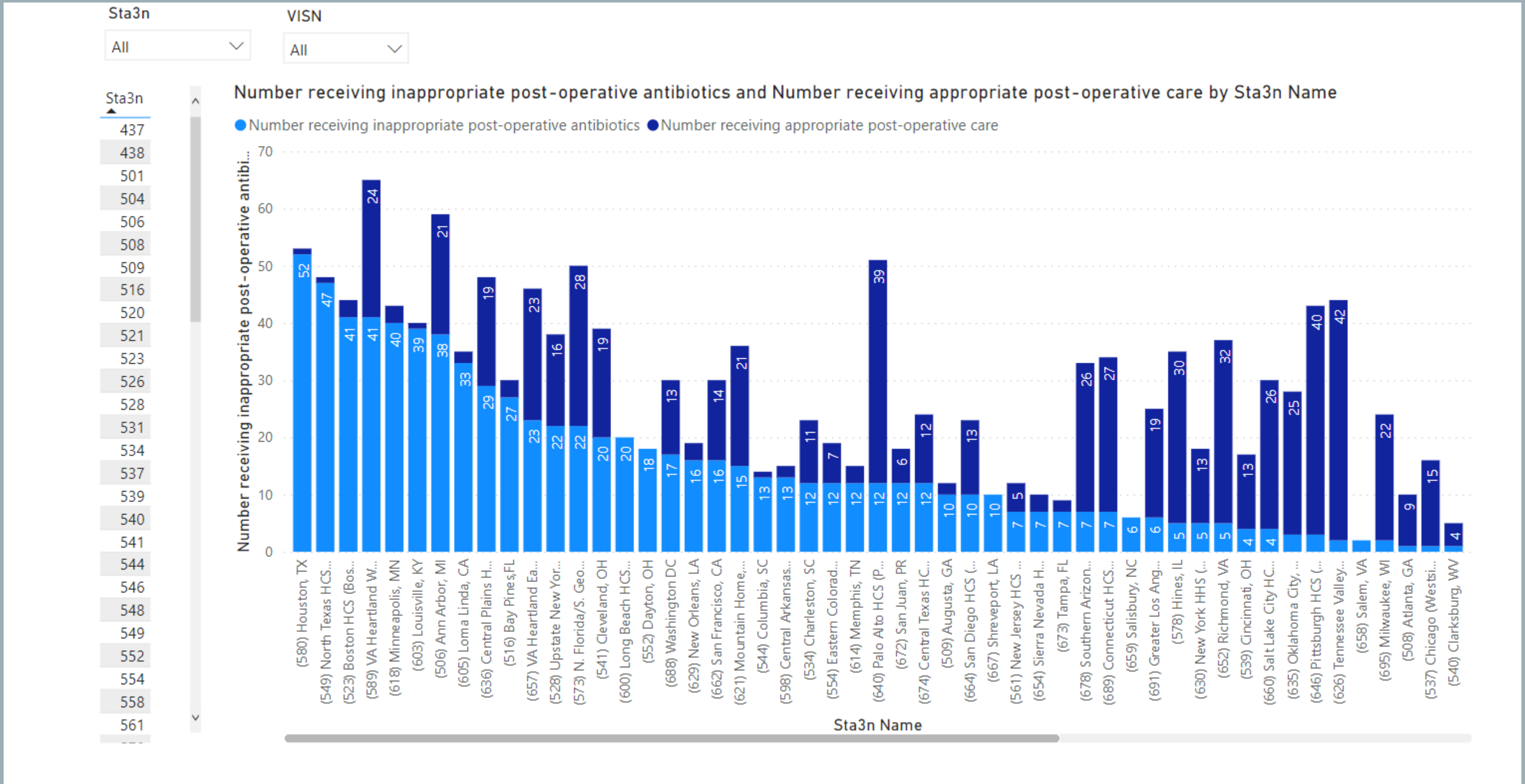
Sta3n
All
VISN
All
Sta3n
437
438
501
504
506
508
509
516
520
521
523
526
528
531
534
537
539
540
541
544
546
548
549
552
554
558
561
Number receiving inappropriate post-operative antibiotics and Number receiving appropriate post-operative care by Sta3n Name
Number receiving inappropriate post-operative antibiotics
Number receiving appropriate post-operative care
Number receiving inappropriate post-operative antibi...
0
10
20
30
40
50
60
70
(580) Houston, TX
(549) North Texas HCS...
(523) Boston HCS (Bos...
(589) VA Heartland W...
(618) Minneapolis, MN
(603) Louisville, KY
(506) Ann Arbor, MI
(605) Loma Linda, CA
(636) Central Plains H...
(516) Bay Pines,FL
(657) VA Heartland Ea...
(528) Upstate New Yor...
(573) N. Florida/S. Geo...
(541) Cleveland, OH
(600) Long Beach HCS...
(552) Dayton, OH
(688) Washington DC
(629) New Orleans, LA
(662) San Francisco, CA
(621) Mountain Home,...
(544) Columbia, SC
(598) Central Arkansas...
(534) Charleston, SC
(554) Eastern Colorad...
(614) Memphis, TN
(640) Palo Alto HCS (P...
(672) San Juan, PR
(674) Central Texas HC...
(509) Augusta, GA
(664) San Diego HCS (...
(667) Shreveport, LA
(561) New Jersey HCS ...
(654) Sierra Nevada H...
(673) Tampa, FL
(678) Southern Arizon...
(689) Connecticut HCS...
(659) Salisbury, NC
(691) Greater Los Ang...
(578) Hines, IL
(630) New York HHS (...
(652) Richmond, VA
(539) Cincinnati, OH
(660) Salt Lake City HC...
(635) Oklahoma City, ...
(646) Pittsburgh HCS (...
(626) Tennessee Valley...
(658) Salem, VA
(695) Milwaukee, WI
(508) Atlanta, GA
(537) Chicago (Westsi...
(540) Clarksburg, WV
Sta3n Name

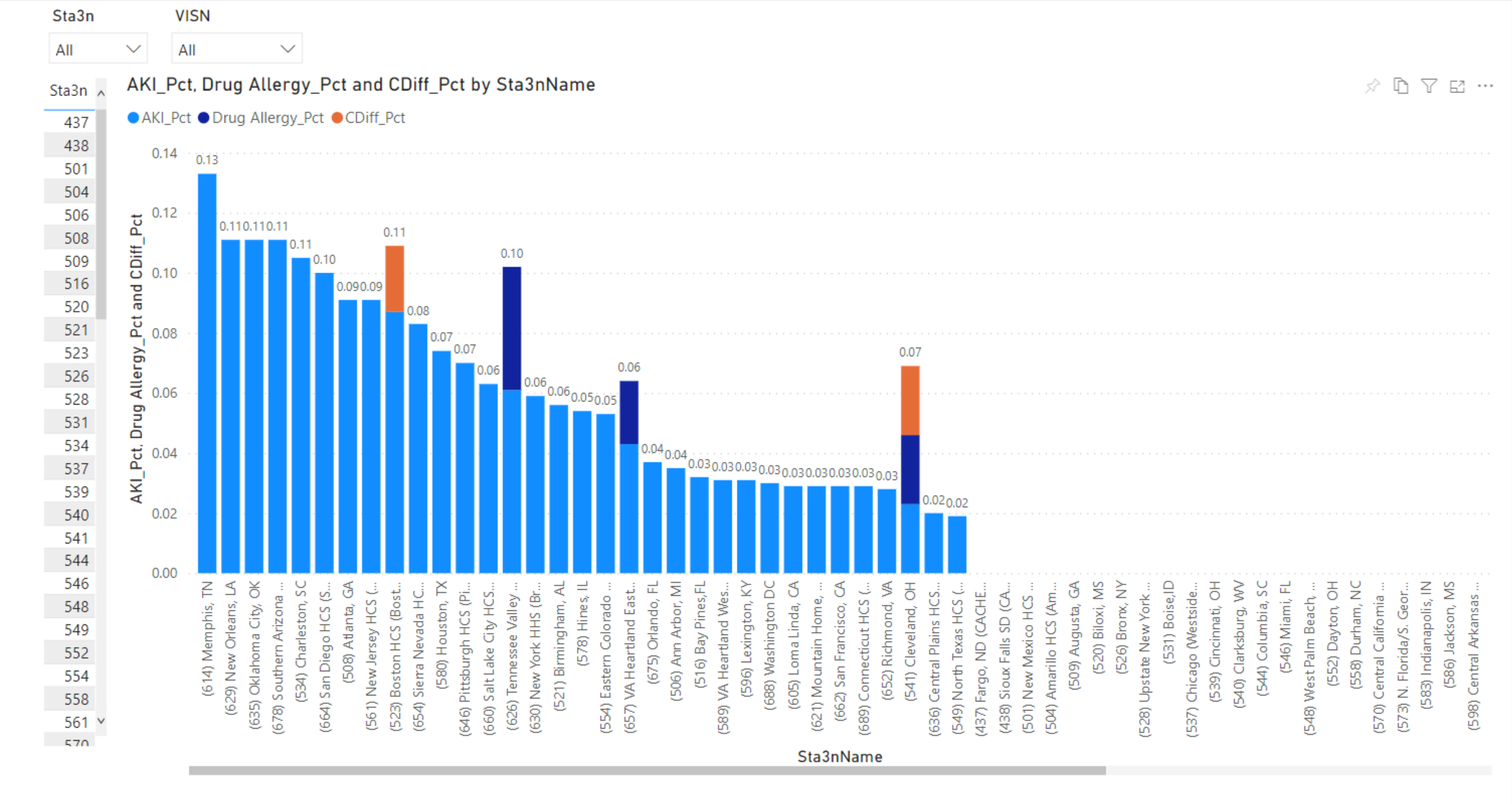

Sta3n
All
VISN
All
Sta3n
437
438
501
504
506
508
509
516
520
521
523
526
528
531
534
537
539
540
541
544
546
548
549
552
554
558
561
AKI_Pct, Drug Allergy_Pct and CDiff_Pct by Sta3nName
AKI_Pct
Drug Allergy_Pct
CDiff_Pct
AKI_Pct, Drug Allergy_Pct and CDiff_Pct
0.14
0.12
0.10
0.08
0.06
0.04
0.02
0.00
0.13
0.11
0.11
0.11
0.11
0.10
0.09
0.09
0.11
0.08
0.07
0.07
0.06
0.10
0.06
0.06
0.05
0.05
0.06
0.04
0.04
0.03
0.03
0.03
0.03
0.03
0.03
0.03
0.03
0.03
0.07
0.02
0.02
(614) Memphis, TN
(629) New Orleans, LA
(635) Oklahoma City, OK
(678) Southern Arizona ...
(534) Charleston, SC
(664) San Diego HCS (S...
(508) Atlanta, GA
(561) New Jersey HCS (...
(523) Boston HCS (Bost...
(654) Sierra Nevada HC...
(580) Houston, TX
(646) Pittsburgh HCS (Pi...
(660) Salt Lake City HCS...
(626) Tennessee Valley ...
(630) New York HHS (Br...
(521) Birmingham, AL
(578) Hines, IL
(554) Eastern Colorado ...
(657) VA Heartland East...
(675) Orlando, FL
(506) Ann Arbor, MI
(516) Bay Pines,FL
(589) VA Heartland Wes...
(596) Lexington, KY
(688) Washington DC
(605) Loma Linda, CA
(621) Mountain Home, ...
(662) San Francisco, CA
(689) Connecticut HCS (...
(652) Richmond, VA
(541) Cleveland, OH
(636) Central Plains HCS...
(549) North Texas HCS (...
(437) Fargo, ND (CACHE...
(438) Sioux Falls SD (CA...
(501) New Mexico HCS ...
(504) Amarillo HCS (Am...
(509) Augusta, GA
(520) Biloxi, MS
(526) Bronx, NY
(528) Upstate New York ...
(531) Boise,ID
(537) Chicago (Westside...
(539) Cincinnati, OH
(540) Clarksburg, WV
(544) Columbia, SC
(546) Miami, FL
(548) West Palm Beach, ...
(552) Dayton, OH
(558) Durham, NC
(570) Central California ...
(573) N. Florida/S. Geor...
(583) Indianapolis, IN
(586) Jackson, MS
(598) Central Arkansas ...
Sta3nName

QUESTIONS?

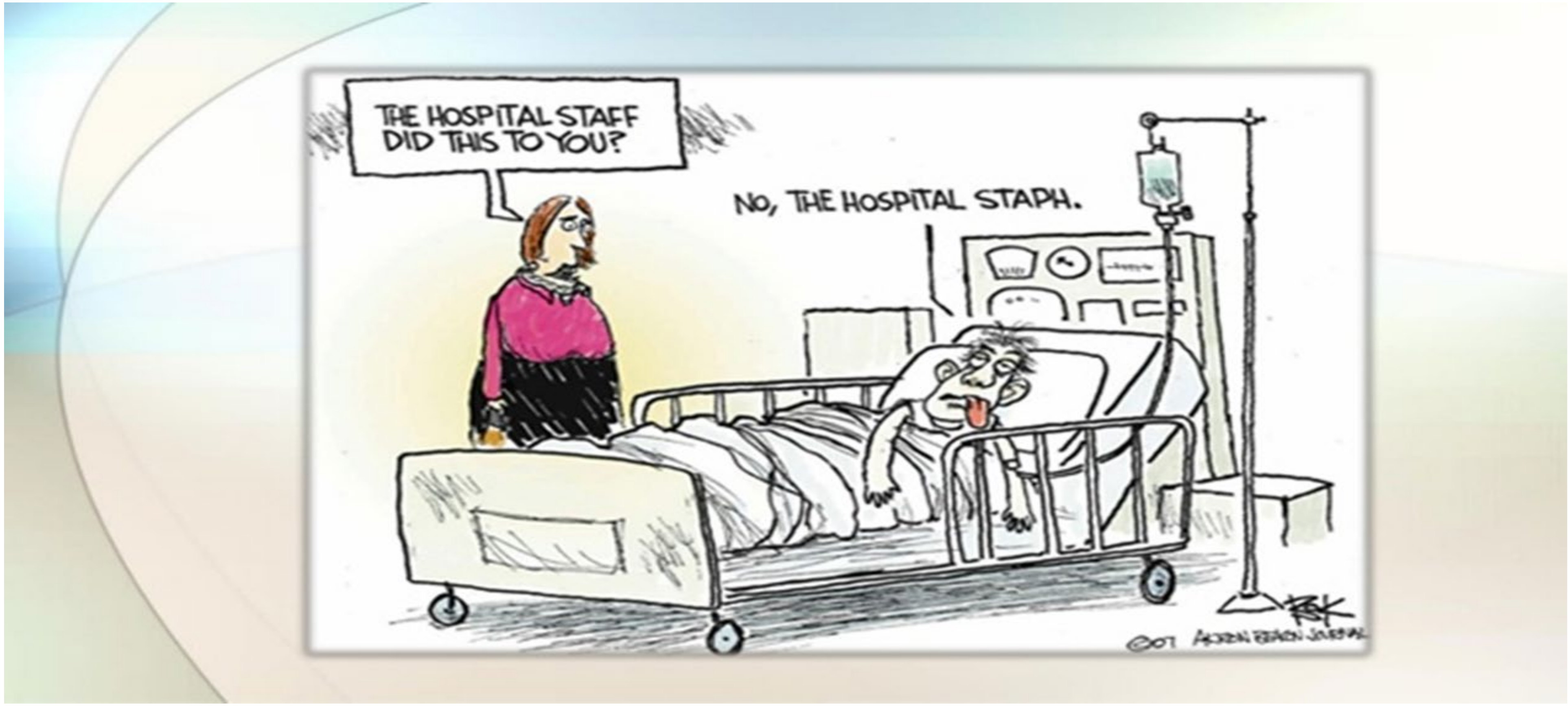

Westyn Branch-Elliman: Westyn.branch-Elliman@va.gov

PUTTING THE PIECES TOGETHER: TOWARD IMPLEMENTATION OF A COMPREHENSIVE QUALITY MONITORING SYSTEM

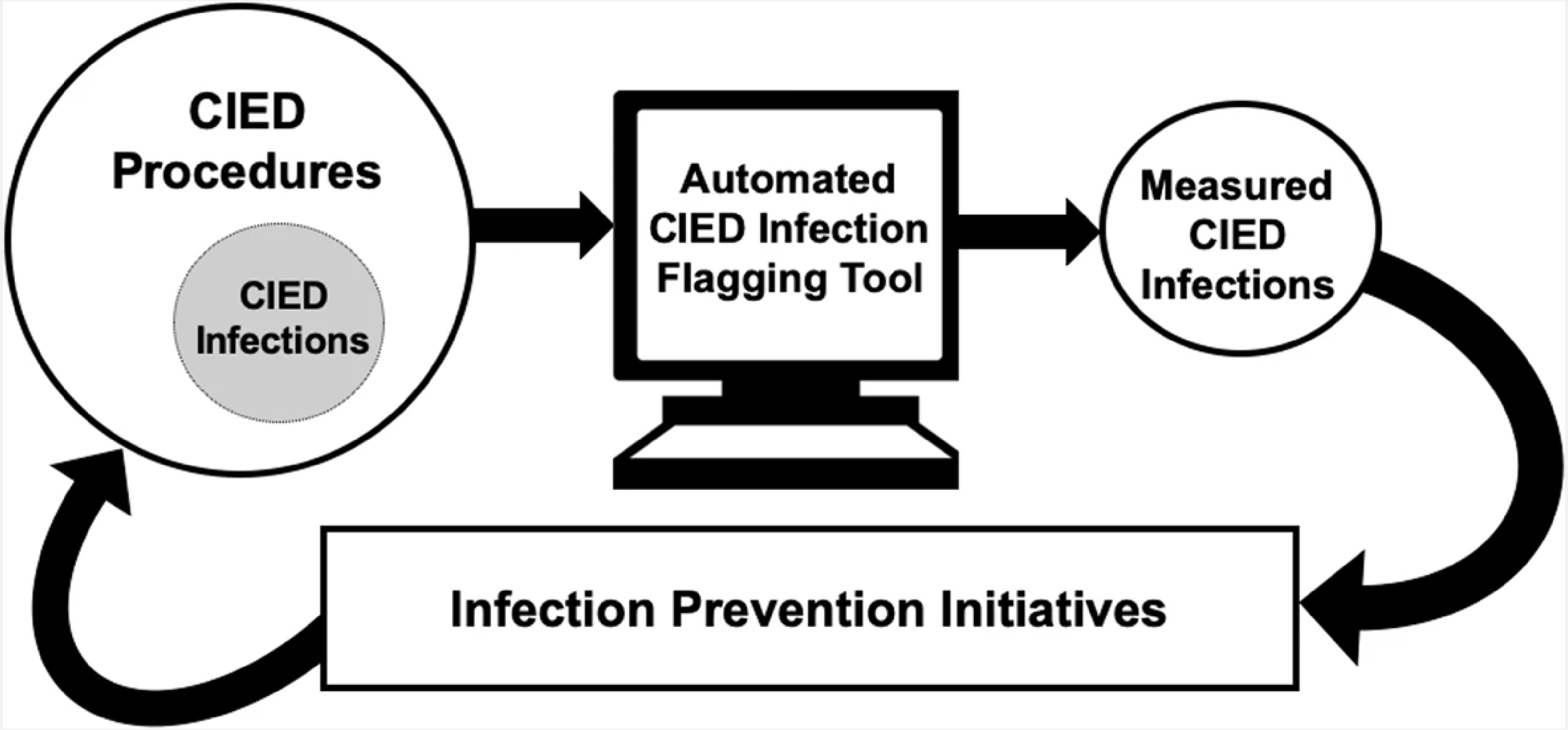

Asundi et al. *Scientific Reports*. 2020

Algorithm Development

Refinement, Validation, and Improving Positive Predictive Value

With clinicians, identify a set of text-strings typically used to document condition of interest

Apply the text string searches to the sub-population with positive flags

Complete a blinded review a sample of cases with positive and negative text string flags, gather examples of flags from clinical notes

Measure sensitivity and specificity of algorithm. Determine if target accuracy is achieved

NO

Identify reasons for failure, including reasons for false positive flags and false negative flags

<u>Adjust algorithm</u>

Change/limit text string searches

Limit flags to specific note types

Change rules for searches (e.g., limit number of spaces between key words)

YES

Algorithm Validation: Measure Sensitivity, Specificity, Positive and Negative Predictive Value

Apply final algorithm to validation dataset: Half of all FY16-17 CIEDs N=9,606 cases

Mull et al. JAMA Open, 2020

WHAT DRIVES USE? HOW CAN WE LEVERAGE QUALITATIVE DATA TO DEVELOP STRATEGIES TO IMPROVE PRACTICE?

We conducted qualitative interviews with electrophysiology providers across the country and gathered data about their current practices

Also sought input on what drives their current practice

Mapped the responses from the qualitative interviews to evidence-based implementation strategies to develop a comprehensive de-implementation plan

		IMPLEMENTATION STRATEGIES
FACILITATORS	Desire to optimize outcomes	Feedback about CIED infections and non-cardiac outcomes (acute kidney injury, *C. difficile* infection)
	Local content experts/opinion leaders	Engagement of local champions to receive and provide audit and feedback reports
	Strength of the data	Provider education
BARRIERS	Lack of resources	Automated, centralized surveillance system
	Difficulty monitoring patients and concern problems would not be identified	
	Lack of knowledge about consequences	Provider education and audit and feedback
	Concern about providing non-standard care	Inclusion of benchmarking, champions to help with development of local policies and procedures

Branch-Elliman et al, AJIC, 2020

HOW CAN WE LEVERAGE THESE SURVEILLANCE SYSTEMS TO IMPROVE PRACTICE? HOW GAPS IN INFORMATION AND FEEDBACK PROMOTE CONTINUATION OF GUIDELINE-DISCORDANT CARE

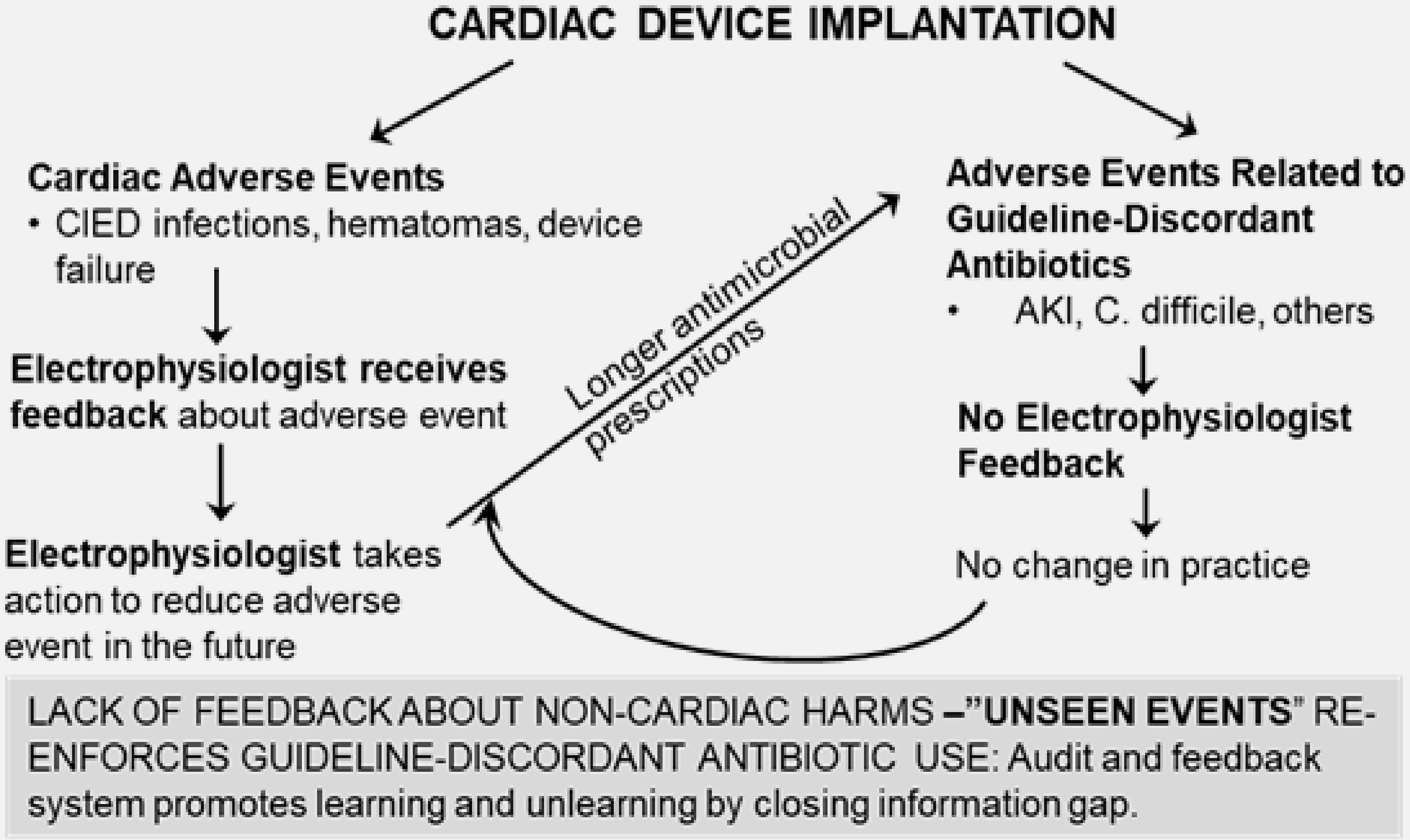

WE KNOW THERE IS A PROBLEM.. HOW CAN WE ADDRESS IT?

Study Aim: Promote the de- adoption of harmful post-procedural antimicrobial use following CIED procedures

Implementation Strategies: Use an innovative informatics system that couples CIED infection monitoring with audit and feedback about compliance with clinical guidelines and other adverse events, facilitation, education

Theoretical Basis: The de-implementation strategy is informed by unlearning and unlearning processes and guided by the iPARIHS framework.

MOVING TOWARDS IMPLEMENTATION

How can these tools be leveraged to change clinical care?

ALGORITHM PERFORMANCE

Sensitivity: 94.4%

Specificity: 48.8%

Development sample

Reviewed 381/9,606 CIED procedures

AUC, 0.93

Among cases with a predicted probability >10%, PPV was 41.2%

Set >10% as the threshold for chart review to operationalize the algorithm

Validation sample

Reviewed 363/9,606 CIED procedures

AUC, 0.90

Predicted probability >10%, PPV 43.5%

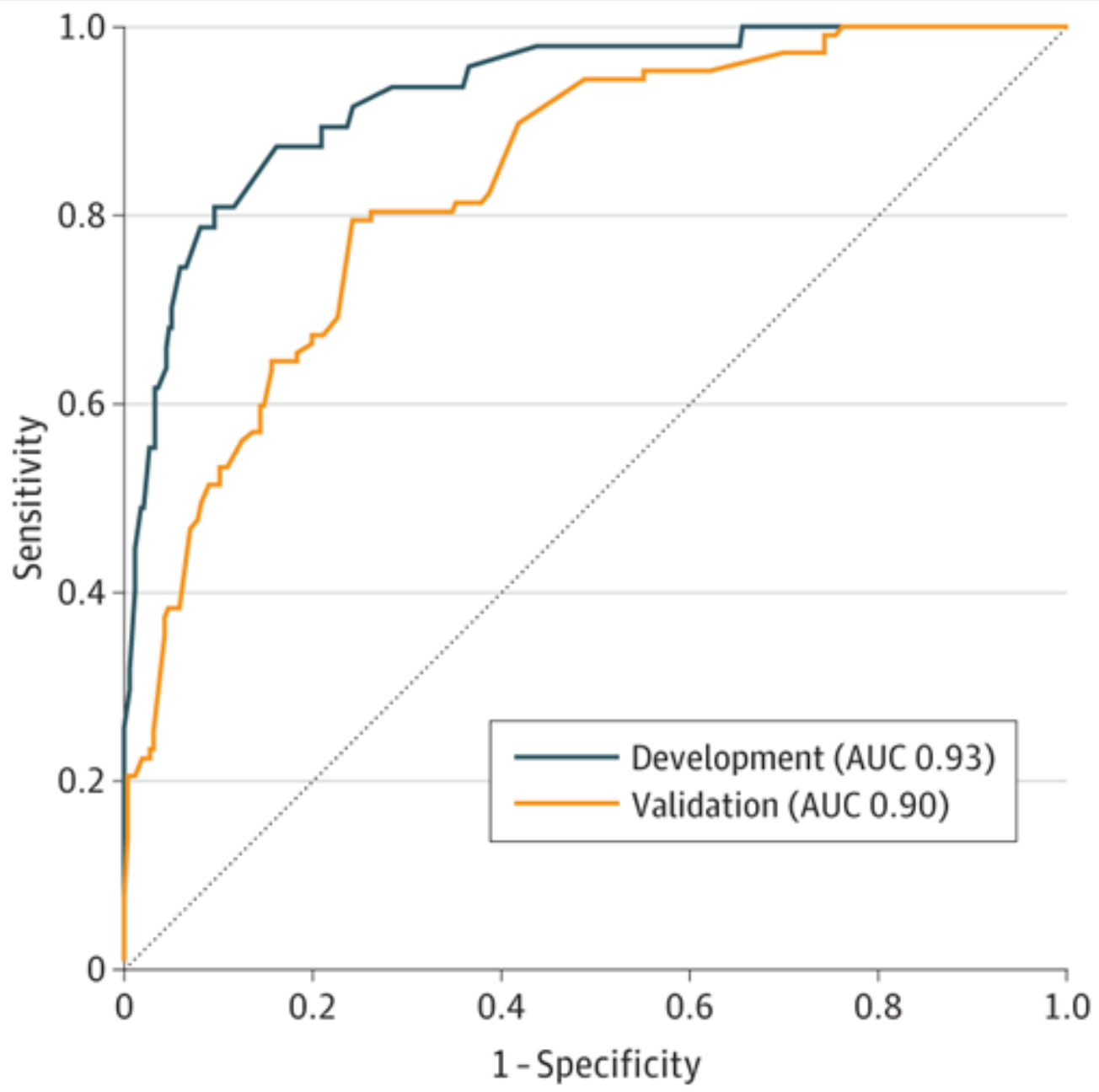

Mull et al. JAMA Open, 2020

CIED PROCEDURES AND POST-PROCEDURAL ANTIMICROBIAL USE

- Unnecessary antimicrobial- related complications occur if antimicrobials are continued for longer than is recommended in clinical guidelines.
- This exposure causes **preventable** harm and contributes to the growing public health crisis of antimicrobial resistance and healthcare-associated infections, which are among the top-ten causes of death in the US
- The **preventable** complications include *C. difficile* infections and acute kidney injuries (AKI), both of which contribute to unnecessary morbidity and mortality.
  - ***C. difficile*** is one of the most common healthcare-associated infection in the US, causing more than 15,000 deaths each year

CHALLENGES AND POTENTIAL SOLUTIONS

- **Fewer than 15%** of facilities nationwide have standardized systems for monitoring compliance with best practices and complications that arise from guideline-discordant care
- Measurement of CIED infections is not standardized within the VA or elsewhere, so there is little infrastructure available for detecting problems when they occur
  - Electrophysiologist report that this is a major driver of practice
- Non-cardiac adverse events are never linked back to their root cause, guideline-discordant antimicrobial use.

PRE-OPERATIVE ANTIMICROBIAL PROPHYLAXIS: EVALUATING REGIMENS

- Current standard-of-care is to give a single agent (Clinical Practice Guidelines developed by the American Society of Health System Pharmacists, IDSA, the Surgical Infection Society, and SHEA)
  - For most surgeries, a beta-lactam is preferred with vancomycin as an alternative in beta-lactam allergic patients.
- Although the standard of care is a single agent, there is mounting evidence that this may not be the optimal strategy for all patients.
  - MRSA colonization status may be a risk-modifier
  - Quazi-experimental study of bundled intervention
- Other studies suggest a potential harm
  - Increased incidence of acute kidney injury in patients receiving dual therapy

Bratzler et al. American Journal of Health-System Pharmacy. Feb 2013.
Gupta et al. ICHE. Aug 2011.
Schweizer et al. JAMA. June 2015.
Stone PA et al. Annals of Surgery. Sept 2015
Courtney PM. Clin Orthop Relat Res. July 2015
