# Supplementary material for: Balanced informational feedback as part of an implementation/deimplementation bundle to support deimplementation efforts in antimicrobial stewardship: results from a mixed methods, hybrid type III implementation/effectiveness trial
Source: Front Public Health. 2026 Sep 23;14:1893058. doi: 10.3389/fpubh.2026.1893058 (PMC13642756; doi:10.3389/fpubh.2026.1893058)
Supplement: Supplementary file 2 [file Data_Sheet_2.PDF]

Power BI Audit/Feedback Dashboard Intervention

Ongoing quality metrics reported in Power BI dashboard and disseminated to sites:

1) Prolonged post-procedural antimicrobial use (≥2 days)
2) Cardiac device (CIED) infections†
3) Antimicrobial-associated harms:
   - Acute kidney injury (AKI), 7d post-procedure
   - *C. difficile* infections, 45d post-procedure
   - Antibiotic allergic events, 45d post-procedure

† *Confirmed via manual review process by study team*

Dashboard: Elements

Post-Procedure Antimicrobial Use

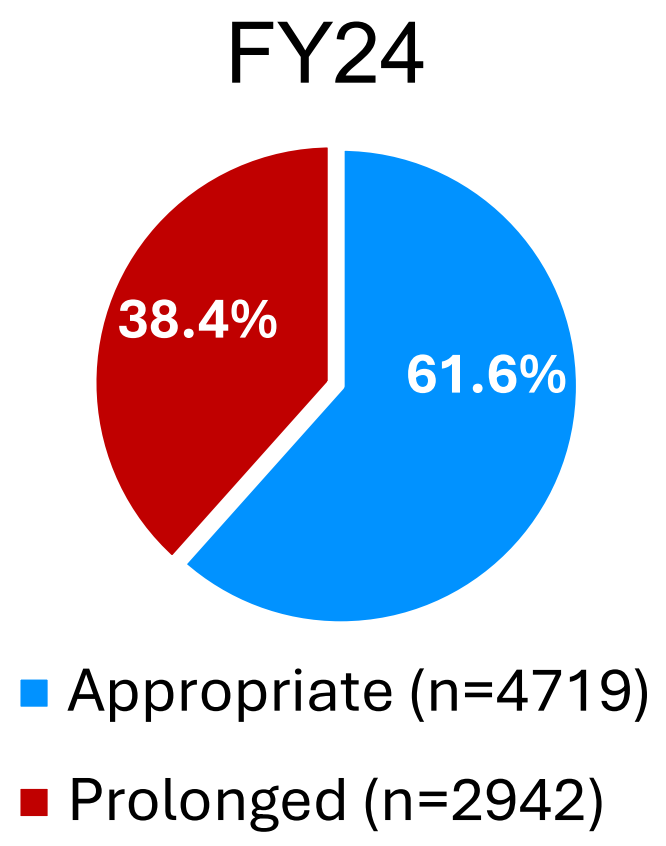

Clinical Outcomes

Clinical outcomes for CIED procedures FY24

Adverse Events	N (%)
Cardiac Device Infection†	33 (0.4)
Acute Kidney Injury	258 (3.4)
C. difficile	124 (1.6)
Allergic Reaction†	12 (0.2)
Total Procedures	*7661*

† Algorithm-predicted infections and allergic reactions are unconfirmed. Denominator for % is total number of patients.

POWER BI Dashboard Live Demo

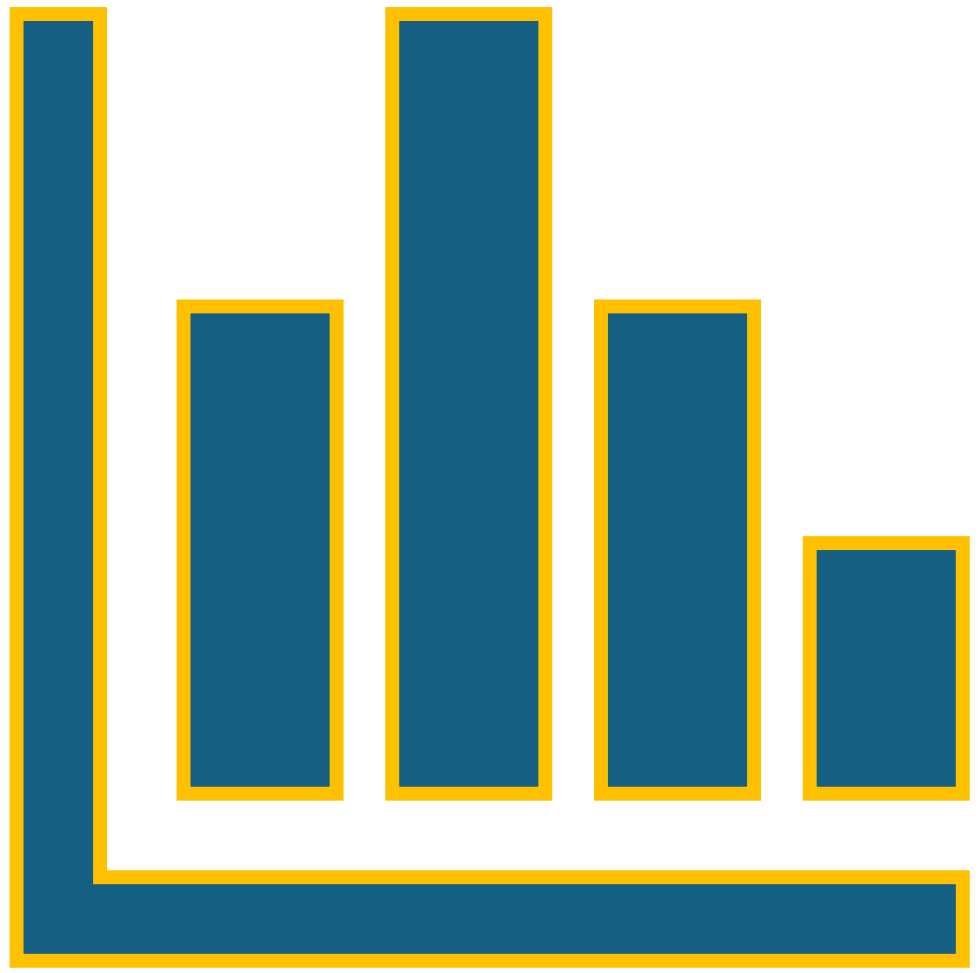

https://app.powerbigov.us/Redirect?action=OpenApp&appId=fb73c209-02db-4414-8ee1-5fd3112fffc5&ctid=e95f1b23-abaf-45ee-821d-b7ab251ab3bf

Data Dictionary

Variable Name	Definition
30_day_mortality_rate	Patient deaths within 30 days of a CIED procedure, reported at the facility level. The numerator is the number of patient deaths and the denominator is the total number of procedures performed.
90_day_mortality_rate	Patient deaths within 90 days of a CIED procedure, reported at the facility level. The numerator is the number of patient deaths and the denominator is the total number of procedures performed.
AKI_Facilityrate_7days	Incidence of Acute Kidney Injury (AKI) during the 7-day surveillance period following CIED procedure at the facility-level using AKIN Network definitions. The numerator is the number of AKIN-defined AKI events and the denominator is the total number of procedures performed. Of note, patients with pre-existing dialysis are excluded from the population for the purposes of calculation of this variable.
Antibiotic_Allergy_Rate	Incidence of antimicrobial-associated allergic reactions during the 45-day surveillance period following CIED procedure at the facility-level using the VA Allergy Reaction Tracking (ART) Package for case ascertainment. The numerator is number of allergic reactions and the denominator is the total number of procedures performed.
CDiff_Facilityrate_90days	Incidence of C. difficile infections (CDI) during the 90-day surveillance period following CIED procedure at the facility-level using microbiologic testing for case ascertainment. The numerator is number of CDI cases and the denominator is the total number of procedures performed.
CIED_InfectionRate_90days_Estimated	Estimated incidence of CIED infection during the 90-day surveillance period following the index CIED procedure at the facility-level. These cases are not manually reviewed. The reported value is based on an electronic algorithm and all cases above a probability threshold of 40% are reported. For this variable, the numerator is the number of patients with a high probability of a true infection and the denominator is the total number of patients undergoing CIED procedures.
Count_AKI_7days	Count of the total number of cases of acute kidney injury (AKI) during the 7-days following a CIED procedure. This variable is electronically derived. Patients receiving hemodialysis prior to their procedure are excluded from this variable.
Count_Allergy	Count of the number of antimicrobial allergic reactions during the 45 days following a CIED procedure. This variable is derived from the Allergy Reaction Tracking (ART) Package in the VA electronic health record. Based on our manual review, we estimate that this health factor has a specificity of 94% and a sensitivity of 63%, thus this is likely an underestimate of the true number of allergic reactions.
Count_Appropriate_Prophylaxis_Duration	Count of the total number of procedures with guideline-concordant antimicrobial use. This count includes all cases during which antimicrobials are discontinued within 24 hours following skin closure.
Count_CDI_90days	Count of the total number of cases of C. difficile infection (CDI) within 90 days following the CIED procedure. This variable is electronically derived and based on microbiologic diagnosis and/or ICD-10 codes for C. difficile infection.
Count_CIEDInfections_90days_PPV40	Count of the number of CIED infections occurring within 90 days of a CIED procedure. This variable is electronically derived and all cases with a predicted

Data Broken Down by Facility and Aggregated

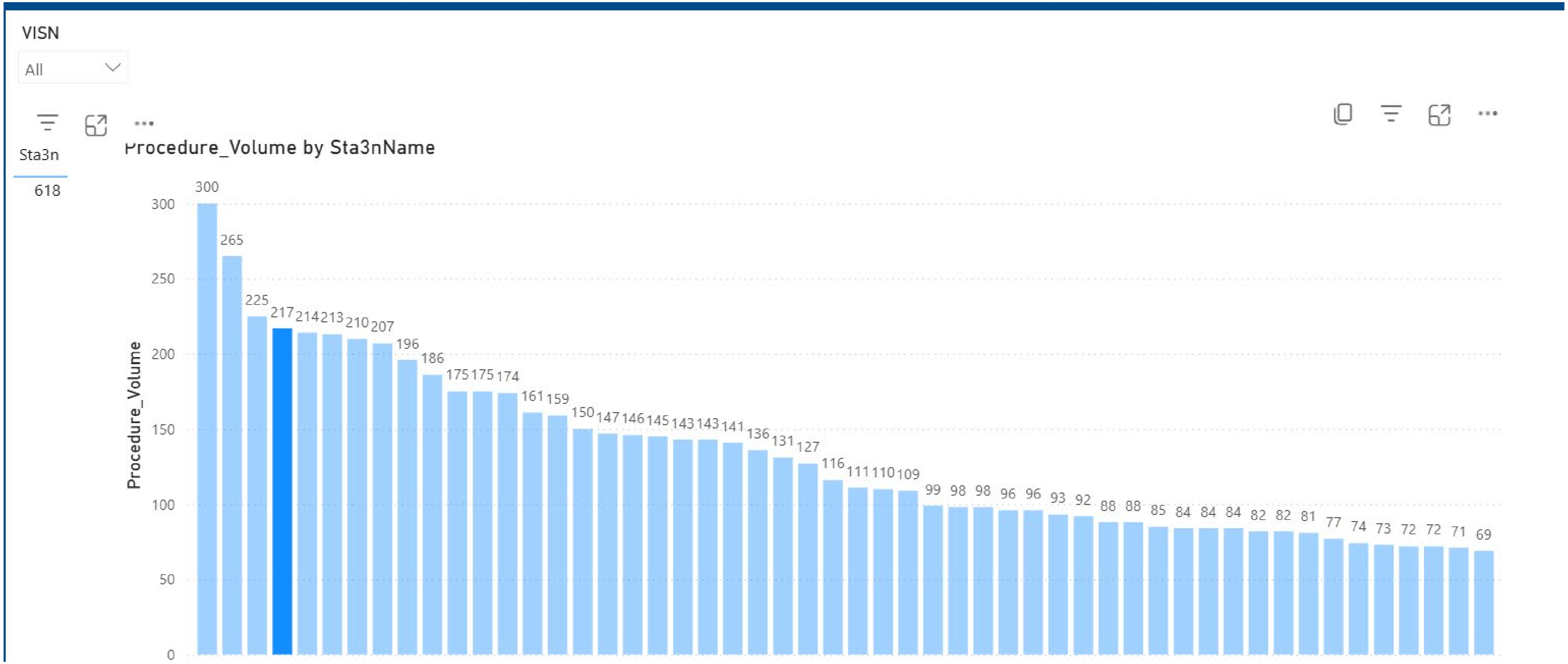

Rates of Guideline-Concordant Prophylaxis Use with National Benchmarking (% Discordant)

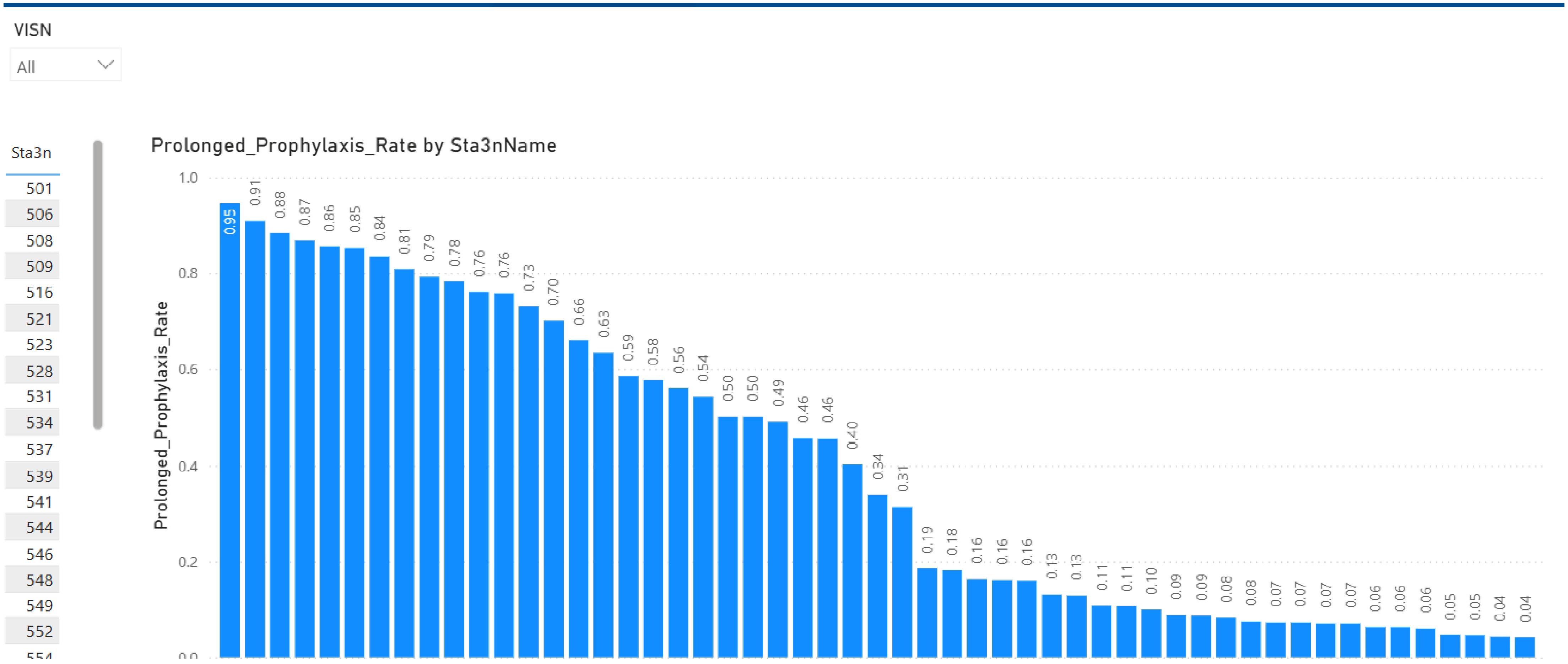

Rates of Guideline-Concordant Prophylaxis Use with National Benchmarking (# Discordant)

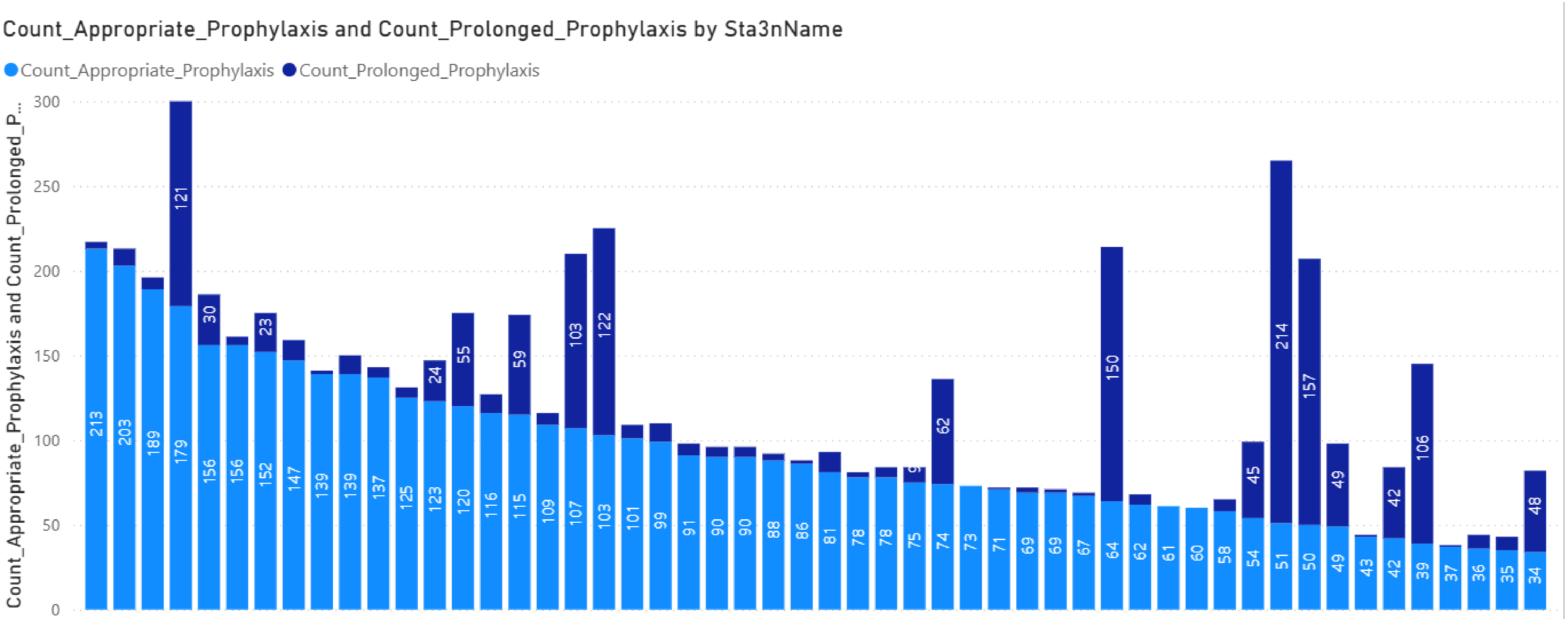

Facility-level CIED infection flagged cases

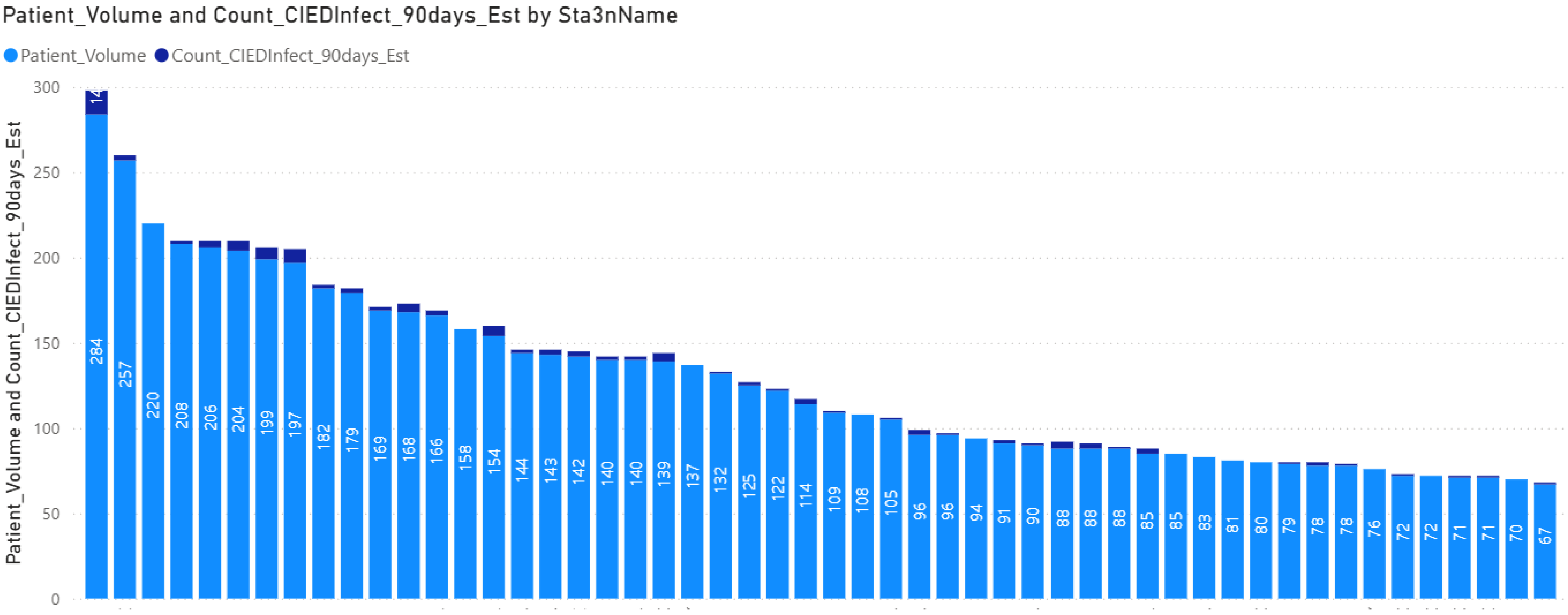

Antimicrobial Adverse Events by Facility

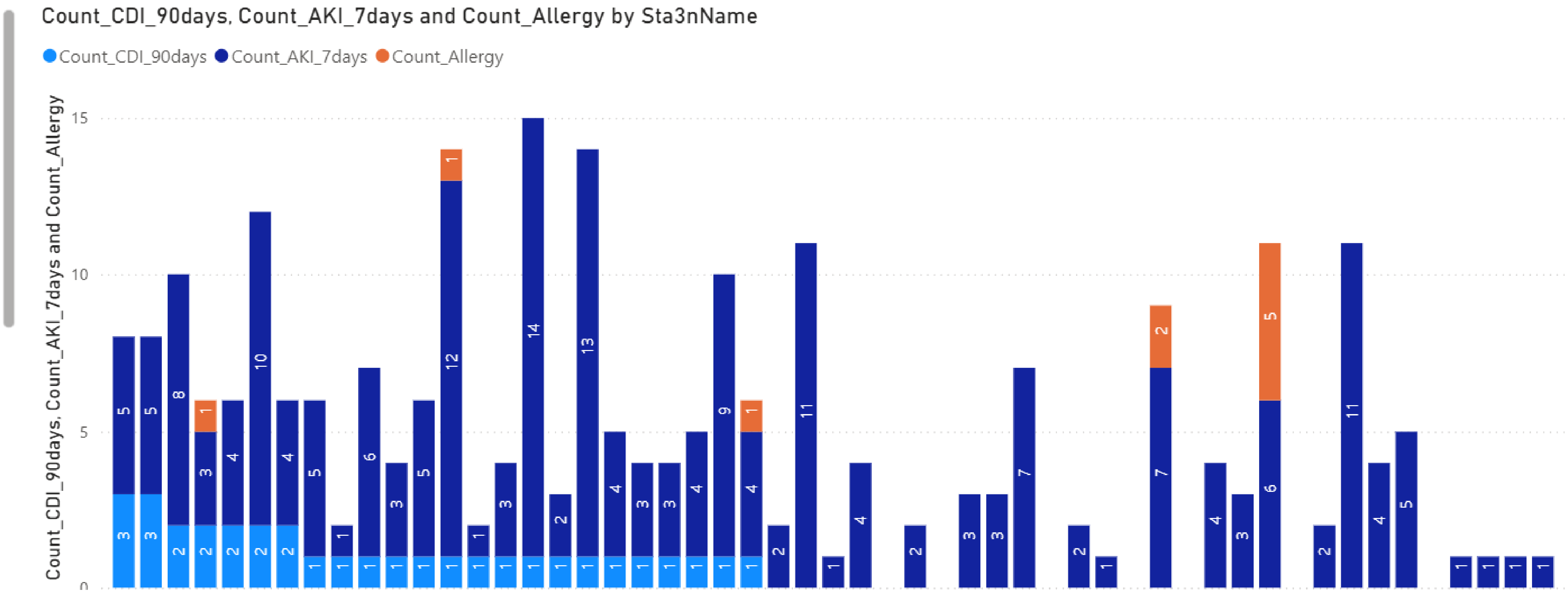

CIED infection rate versus prolonged antibiotic use

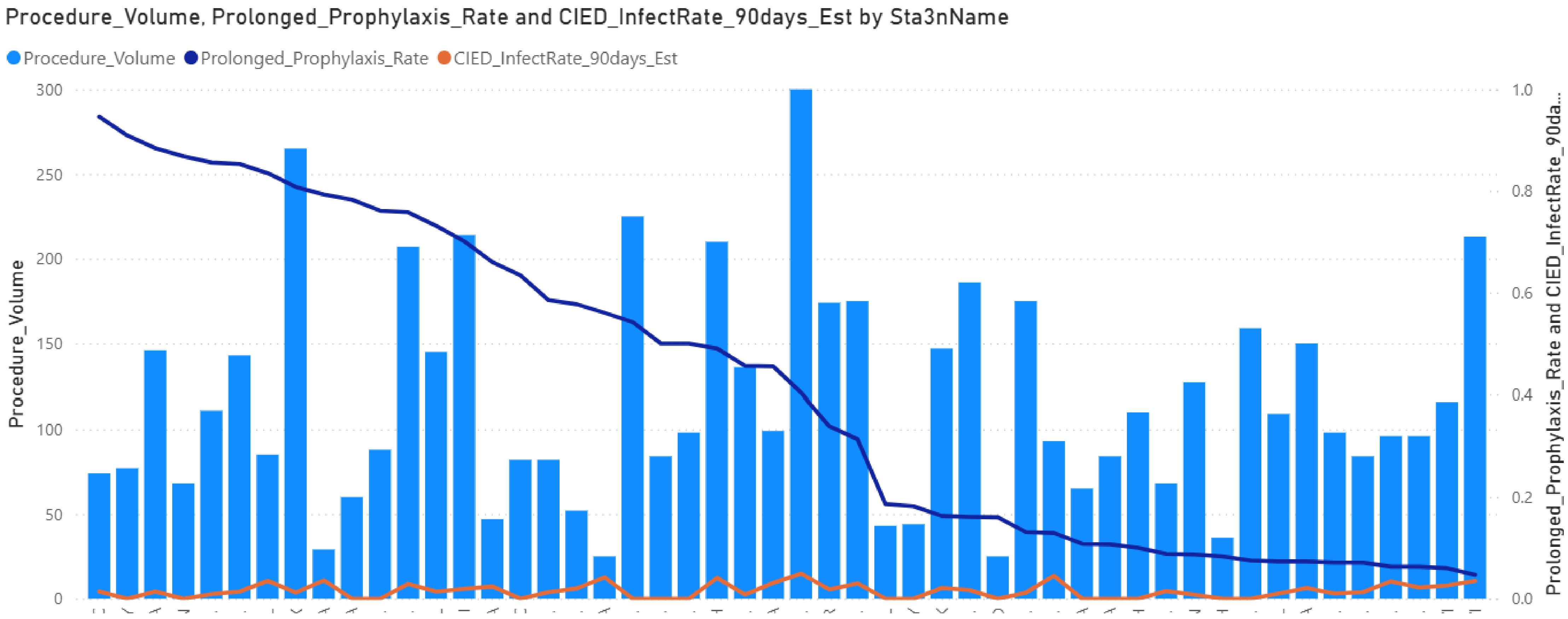

Report with PHI

VISN: All

Sta3n: All

PredInfection Flag: All

AKI: All

CDiff: All

ObservedAllergy_Flag: All

Died90: All

Discordant PostOpAbx_Flag: All

TotalAbxDays: 0 – 9

36

Count of PatientSSN

ProcedureDate: 10/4/2022 – 12/28/2022

Sta3nName	PatientSSN	PatientLastName	PatientFirstName	ProcedureDate	Discordant PostOpAbx_Flag	TotalAbxDays	AKI	CDiff	ObservedAllergy_Flag	Died90	PredInfection Flag
(523) Boston HCS				12/15/2022	1	6	0	0	0	0	0
(523) Boston HCS				11/29/2022	1	5	0	0	0	0	0
(523) Boston HCS				12/13/2022	1	9	1	1	0	0	0
(523) Boston HCS				12/16/2022	1	6	1	1	0	0	0
(523) Boston HCS				12/19/2022	1	4	0	0	0	0	0
(523) Boston HCS				10/17/2022	1	4	0	0	0	0	0
(523) Boston HCS				12/22/2022	1	5	0	0	0	0	0
(523) Boston HCS				10/4/2022	1	5	0	0	0	0	0
(523) Boston HCS				11/2/2022	1	5	0	0	0	0	0
(523) Boston HCS				11/1/2022	1	5	0	0	0	0	0
(523) Boston HCS				11/3/2022	1	4	0	0	0	0	0
(523) Boston HCS				12/15/2022	1	2	0	0	0	0	0
(523) Boston HCS				12/22/2022	1	5	0	0	0	0	1
(523) Boston HCS				10/27/2022	1	6	0	0	0	0	0
(523) Boston HCS				10/28/2022	1	4	0	0	0	0	0
(523) Boston HCS				10/6/2022	1	5	0	0	0	0	0
(523) Boston HCS				10/21/2022	1	4	0	0	0	0	0
